# N3A motifs in RIβ mediate allosteric crosstalk between cAMP and ATP in PKA activation

**DOI:** 10.1002/pro.70332

**Published:** 2025-10-18

**Authors:** Jian Wu, Jessica G. H. Bruystens, Puspashree Sahoo, José Bubis, Rodrigo A. Maillard, Susan S. Taylor, Ronit Ilouz

**Affiliations:** ^1^ Department of Pharmacology University of California at San Diego San Diego California USA; ^2^ The Azrieli Faculty of Medicine Bar Ilan University Safed Israel; ^3^ Departamento de Biología Universidad Simón Bolívar Caracas Venezuela; ^4^ Dirección de Salud, Fundación Estudios Avanzados IDEA Caracas Venezuela; ^5^ Department of Chemistry Georgetown University Washington, DC USA; ^6^ Institute for Soft Matter Synthesis and Metrology Georgetown University Washington, DC USA; ^7^ Department of Biochemistry and Molecular Biophysics University of California at San Diego San Diego California USA; ^8^ The Leslie & Susan Goldschmied (Gonda) Multidisciplinary Brain Research Center Bar‐Ilan University Ramat‐Gan Israel

**Keywords:** heterodimer structure, holoenzyme, PKA, PRKAR1B

## Abstract

The RIβ subunit of cAMP‐dependent protein kinase (PKA) is highly expressed in the brain, yet it remains the least studied of the PKA regulatory subunits (R). As pathologic variants of its gene are increasingly implicated in neurodevelopmental disorders, neurodegeneration, and cancer, gaining more information about the structure/function of RIβ, and how it differs from RIα, has become increasingly important. We previously reported the structure of the RIβ_2_C_2_ holoenzyme, which revealed a novel conformation where ATP binding was stabilized by a head‐to‐head anti‐parallel packing of the C‐tail wrapped around the N‐lobe of the catalytic subunit (C). Although visible, the Dimerization/Docking Domain was poorly folded and reduced. Since RIβ is oxidized in brain tissues, we asked if oxidation or binding of an A Kinase Anchoring Protein (AKAP) would affect the holoenzyme structure. Oxidation or addition of an AKAP peptide to crystals led to the release of nucleotide. To capture this at higher resolution we crystallized RIβ_2_C_2_ in the presence of an AKAP peptide. This new structure represents an RIβ:C heterodimer. Density for the D/D domain was missing; ATP was absent, the kinase adopted an open conformation, and the C‐terminus of the RIβ subunit was no longer resolved. Because the crosstalk between ATP and cAMP in the R:C complex appears to be mediated by the two N3A motifs (N3A^A^ and N3A^B^) as well as by the linker, which in free RIβ is intrinsically disordered, we describe the conserved features of these two motifs as well as the linker and show how each contributes in a unique but coordinated way to allosteric activation of RIβ holoenzymes by cAMP. A key difference in our RIβ:C structure is the rotation of the side chain of W260 at the N‐terminus of the αA Helix in N3A^B^. W260, at the R:C interface in the holoenzyme, is also the capping residue for cAMP bound to CNB‐A, so we may have actually captured the first step in cAMP activation.

## 1 INTRODUCTION

The catalytic (C) subunit of cAMP‐dependent protein kinase (PKA) was the second protein kinase to be discovered (Walsh et al. 1968), and the PKA regulatory (R) subunits that bind cAMP and package the C‐subunits into an inactive R_2_C_2_ holoenzyme were discovered shortly thereafter (Reimann et al. 1971). A simple paradigm for cAMP activation of the PKA holoenzyme was thus established early on, including the fundamental differences between RI and RII isoforms (Erlichman et al. 1974; Walsh et al. 1971). These R‐subunits are functionally non‐redundant (Cadd and McKnight 1989), and although they all have the same general domain organization that includes an N‐terminal docking and dimerization (D/D) domain, a flexible linker, and two tandem cyclic nucleotide binding (CNB) domains (CNB‐A and CNB‐B) at the C‐terminus, the quaternary structures of each holoenzyme are distinct, as are the allosteric networks that link cAMP binding to PKA activation (Taylor et al. 2012). Each CNB domain begins with the N3A motif, which is a conserved structural element consisting of an N‐terminal helix, an eight‐residue 3^10^ loop, and the A‐helix (N3A). Another class of proteins that contribute significantly to PKA signaling and specificity are the A Kinase Anchoring Proteins (AKAPs) that target PKA to specific sites in the cell (Pawson and Scott 2005), which we referred to as “PKA Signaling Islands.” These PKA scaffold proteins are highly diverse, but all contain a small amphipathic helix that targets the PKA holoenzyme to specific sites by binding with high affinity to the D/D domains of the R‐subunits (Newlon et al. 1999). Most AKAPs are specific for RII‐subunits, but some are dual‐specific (Huang et al. 1997) and a few, such as the smAKAP (Burgers et al. 2012) and the cilia‐specific GPR161 (Bachmann et al. 2016), are RI‐specific. The combinatorial features introduced by the R‐ and C‐subunit isoforms and AKAPs thus make PKA signaling considerably more complex than was originally anticipated or appreciated.

In mammalian cells, separate genes encode four different PKA R‐subunits (RIα, RIβ, RIIα, and RIIβ). Depletion of each R‐subunit isoform displays a unique phenotype, confirming that they are functionally non‐redundant (Cadd and McKnight 1989). Crystal structures of three holoenzymes indicate that the quaternary structures of the isoforms are also distinct, as are the allosteric pathways that lead to activation (Taylor et al. 2012). RIβ is the least studied of the PKA holoenzymes although depletion of RIβ gives a distinct neuronal phenotype (Cadd and McKnight 1989), and recent disease mutations linked to RIβ show neurodegenerative phenotypes as well as neurodevelopmental disorders (Benjamin‐Zukerman et al. 2025; Benjamin‐Zukerman et al. 2024; Marbach et al. 2021). RIβ is also highly enriched in the brain and localized to the hippocampus (Ilouz et al. 2017), which is consistent with learning defects when RIβ is depleted. Like RIα, RIβ is a pseudo‐substrate in contrast to RIIα/RIIβ, which are both PKA substrates. Here we explore a new RIβ:C heterodimer structure and compare it to RIα holoenzymes and to the full‐length RIβ_2_C_2_ holoenzyme.

Our initial structure of the R_2_C_2_ RIβ holoenzyme showed a distinct conformation that was different from the RIIβ holoenzyme (Zhang et al. 2012), the modeled structure of the RIα holoenzyme (Boettcher et al. 2011) and our subsequent structures of R_2_C_2_ RIα holoenzymes (Lu et al. 2019). Two features were unusual in that initial RIβ structure (Figure S1, Supporting Information). First, the D/D domain in RIβ was visible but appeared to be reduced, poorly ordered, and located under the C‐lobe of the C‐subunit. Second, the C‐terminal tail in the C‐subunit formed an anti‐parallel dimer with the C‐tail of the opposite C′‐subunit that most likely contributed to a stable ATP binding site. A sequence alignment of human RIα and RIβ highlighting the domain organization and elements of secondary structure is summarized in Figure S1. While the RII‐subunits separated early in evolution from the RI‐subunits, the RIα and RIβ diverged much more recently (Canaves and Taylor 2002).

Because RIβ appears to be disulfide bonded in brain tissues (Benjamin‐Zukerman et al. 2024), we asked if changes in the D/D domain, either by oxidation or binding of an AKAP peptide, lead to changes in the R_2_C_2_ structure solved previously. Because oxidation or adding an AKAP peptide caused the holoenzyme crystals to dissolve, we added a RI‐specific AKAP peptide (smAKAP) to the full‐length holoenzyme and co‐crystalized the complex. The resulting protein had proteolyzed and led to a new N3A:N3A′ dimer interface involving the N3A motif in CNB‐A, a motif that we have seen previously in the full‐length RIα dimer. In addition, the nucleotide was missing, which leads to a more open and dynamic N‐lobe of the C‐subunit. Finally, the C‐terminal C′/C″ helix motifs in RIβ were unresolved, which interferes with coupling to the second N3A motif in CNB‐B. This structure provides new insights into the allosteric network that inversely couples the binding of ATP with the binding of cAMP, and correlates this with the intrinsically disordered linker where the two ends that flank the inhibitor site contribute to the ordering of ATP to the N‐lobe (N‐linker) and regulating cAMP binding to the C‐lobe (C‐linker). The unique features of the highly stable N3A motifs, one in CNB‐A (N3A^A^) and the other in CNB‐B (N3A^B^), are also defined as stable polyvalent hubs that mediate multi‐valent allosteric crosstalk between cAMP and ATP. Finally, we define W260, which lies in the αA helix of CNB‐B and is conserved in both RIα and RIβ, but not in RII subunits, as an essential sensor of cAMP binding that contributes to the activation of the holoenzyme.

## 2 RESULTS

### 2.1 Effect of AKAP binding on the RIβ holoenzyme crystals

Recent studies indicate that in brain tissues RIβ is disulfide bonded in the cytoplasm (Benjamin‐Zukerman et al. 2024), and we confirmed here that RIβ is highly expressed in brain tissues compared to other tissues and that it is disulfide bonded (Figure S2). In our original structure of the RIβ holoenzyme, the dimerization domain was reduced and the density that we attributed to the dimerization domain was not folded into a stable four‐helix bundle although much of the backbone could be traced (Figure S1) (Ilouz et al. 2012). To test whether folding of the D/D domain would influence the crystal packing, we first added diamide to the crystals, which induces the interchain disulfide bonding of the D/D domain (León et al. 1997). The crystals were shattered under these conditions. As an alternative strategy, we then added a small RI‐specific AKAP peptide derived from the smAKAP (TVILEYAHRLSQDILCDALQQWAC) (Burgers et al. 2012; Burgers et al. 2016). The consequences of adding the AKAP peptide to the crystal were several and could be captured, albeit at low resolution. First, the density for the predicted D/D domain disappeared. Second, the density for the nucleotide was lost. Third, the C‐subunit seemed to assume a more dynamic state with the C‐tail becoming less ordered. The original crystals before soaking only diffracted to 3.7 A. Given the even lower resolution of the soaked crystals, we decided to co‐crystallize the RIβ holoenzyme with the smAKAP peptide.

### 2.2 Structure of the RIβ:C heterodimer

**FIGURE 1:**
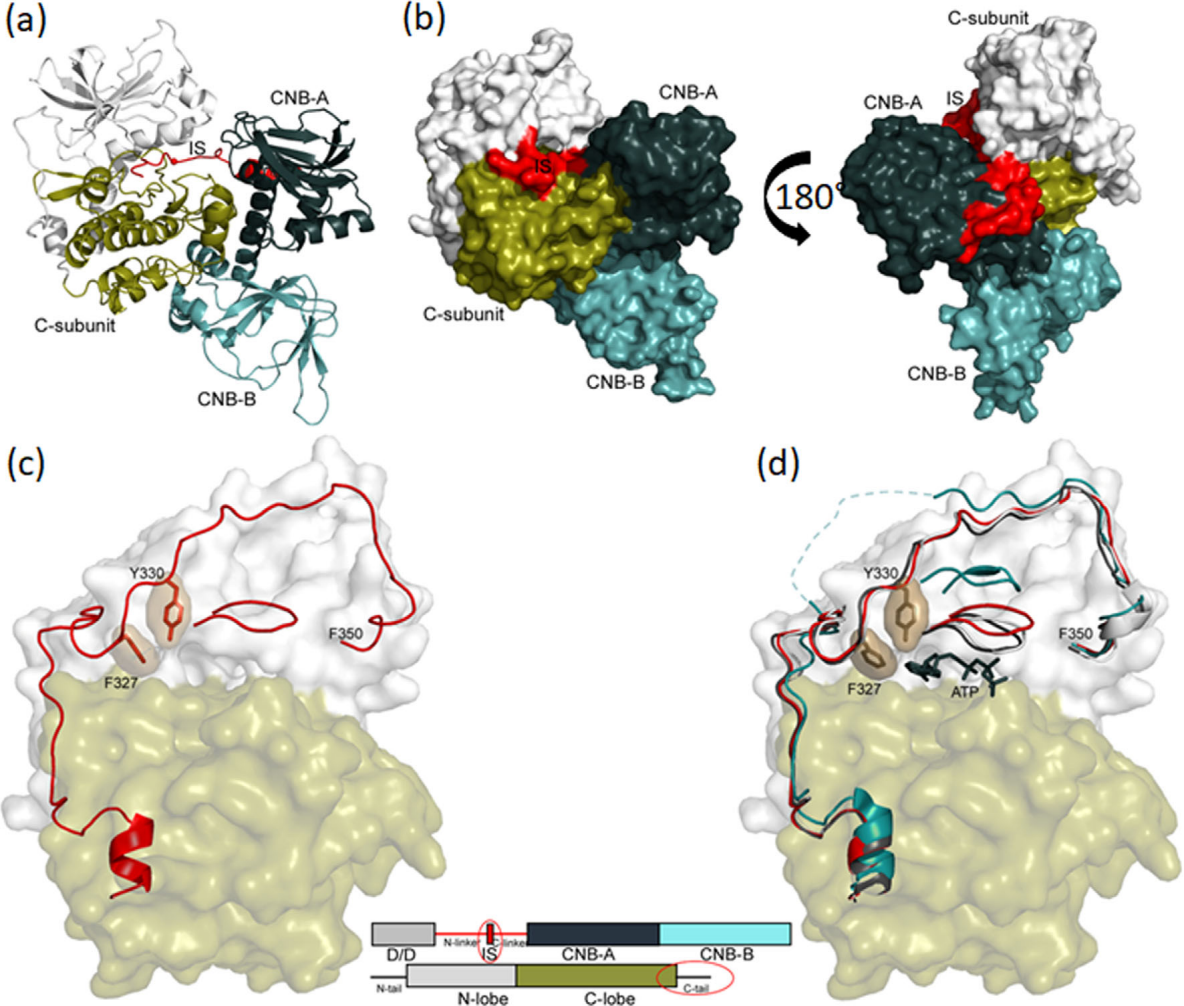
Overall structure of RIβ:C heterodimer. (a) Cartoon representation of the RIβ:C heterodimer structure. (b) Surface representation of the RIβ:C heterodimer structure, with a 180° rotation on the far right. The color coding is the same as (a). (c) G‐loop is in a partially open conformation, and the ATP is missing in RIβ:C structure. The C‐subunit are shown in a surface representation; N‐lobe is in white and C‐lobe in tan. The C‐tail, the residues T300 to F350, and the G‐loop are shown in red. Two key residues F327 and Y330 are highlighted. (d) Comparison of the G‐loop in RIβ:C (red) with a fully closed (dark blue, PDB: 1ATP) and open (cyan, PDB: 1J3H) conformations. The same color coding was applied for all the following figures: The N‐ and C‐lobe of C‐subunit are shown in white and tan, the CNB‐A and CNB‐B domains of RIβ are in dark blue and light blue, the Inhibitor site in red, the PBC motif in golden, the Linker in dark yellow, αB/C/N helix in dark red, respectively. To highlight the main focus of each figure, we also colored those elements in red while keeping the other elements in this color code.

Although we set up full‐length R_2_C_2_ holoenzyme plus the AKAP peptide for co‐crystallization, the density map corresponded to a heterodimer where the R‐subunit had been cleaved prior to the inhibitor site (Figure 1, and the bold black box in Figure S1); that is, no density was observed for the D/D domain or the AKAP peptide. Gel electrophoresis of the crystal confirmed that the full‐length RIβ had been cleaved to a 38 kDa fragment (Figure S3).

This structure of the truncated RIβ subunit bound to the C‐subunit, solved at 3.7 Å (Table S1), reflects the general quaternary organization that is seen in the other R:C complexes, including RIα (Kim et al. 2005; Kim et al. 2007), RIIα (Wu et al. 2007), RIIβ (Brown et al. 2009), and the R_2_C_2_ RIβ holoenzyme (Ilouz et al. 2012). Several differences, however, are apparent in both the C‐subunit and the RIβ subunit compared to the RIβ_2_C_2_ structure solved previously without the AKAP peptide. The absence of ATP at the cleft of the C‐subunit suggests that the N‐lobe is more dynamic and in an open‐like conformation while in the RIβ subunit density is missing for the C‐terminal helix in CNB‐B. We focus first on changes in ATP binding to the N‐lobe of the C‐subunit and then turn our attention to the intrinsically disordered linker in the RIβ subunit. Lastly, we describe the two N3A motifs and, in particular, show how the N3A^B^ motif in CNB‐B allosterically controls access of cAMP to the C‐lobe of the C‐subunit. Finally, we show how W260 and the flexibility of the C‐terminal tail in the RIβ:C complex contribute to the finely tuned signaling message that is transmitted to CNB‐A when cAMP binds to CNB‐B and initiates activation of the holoenzyme. The strategic differences between RIα and RIβ are also identified.

Table S2 summarizes some of the key functional residues in the two RIβ structures and also highlights residues that are solvent exposed. Some side chains play important roles in both the holoenzyme and in the cAMP‐bound conformation, but many are functionally important in one structure and solvent exposed in the other.

### 2.3 N‐lobe and ATP binding site in the catalytic subunit

Although we see density for backbone residues of the G‐Loop and the DFG motif, there is no density for ATP in the active site cleft (Figure S4). The G‐Loop is also in a partially open conformation, and multiple additional features indicate that this N‐lobe is in a more dynamic state compared to the closed conformations described previously for RIα R:C complexes and for the earlier R_2_C_2_ RIβ holoenzyme (Figure 1c,d). Specifically, no density is seen for Phe54 at the tip of the G‐Loop. When the C‐subunit is in a fully closed conformation, this Phe reaches over to Phe187 following the DFG motif in the C‐lobe and forms a hydrophobic shell around the phospho‐transfer site. Although backbone residues of the C‐terminal tail, residues 319–350, can be traced, the density for many of the side chains is poorly resolved or missing (Figure S4). Although density is observed for Phe327 and Tyr330, which are both part of the adenine binding pocket, it is weak. The hydrophobic motif (HM), specifically Phe347 and Phe350, at the C‐terminus anchors the C‐tail to the αC helix. Density is seen for Phe347; however, no density is seen for the C‐terminal Phe350, which also is part of the usually stable and allosterically important HM anchor to the N‐lobe (Arencibia et al. 2013). Other side chain density is notably missing for residues that anchor the C‐terminal tail onto the N‐lobe in the closed conformation. This includes Asp333 in the C‐tail and Arg56 in the G‐Loop, which typically form a stable electrostatic interaction. Finally, the density for Lys72, the highly conserved residue that anchors β strand 3 to the conserved glutamate (Glu91) in the αC helix, is also missing. Typically, the methylene groups of Lys72 are anchored rigidly to hydrophobic residues in the beta sheet of the N‐lobe. Although the resolution is only 3.7 Å, all these observations support the general conclusion that the N‐lobe of the C‐subunit is in a highly dynamic and partially open state, which likely explains why the nucleotide is missing. The lack of density for key hydrophobic residues, in particular, suggests that the N‐lobe is highly destabilized.

### 2.4 Linker in RIα and RIβ is intrinsically disordered

Although we do not have a structure of the cAMP‐bound RIβ dimer, structures of RIα and RIIβ show that much of the linker region that follows the D/D domain and extends to the N‐helix of the CNB‐A domain is an intrinsically disordered region (IDR) that only becomes ordered when the R‐subunit is bound to the C‐subunit. The Inhibitor Site (IS), which docks into the active site cleft, is embedded in the disordered linker. The IS is flanked by N‐ and C‐terminal linkers that reach, respectively, to the ATP binding site in the N‐lobe and control access to cAMP in the C‐lobe.

#### 2.4.1 Inhibitor binding site

**FIGURE 2:**
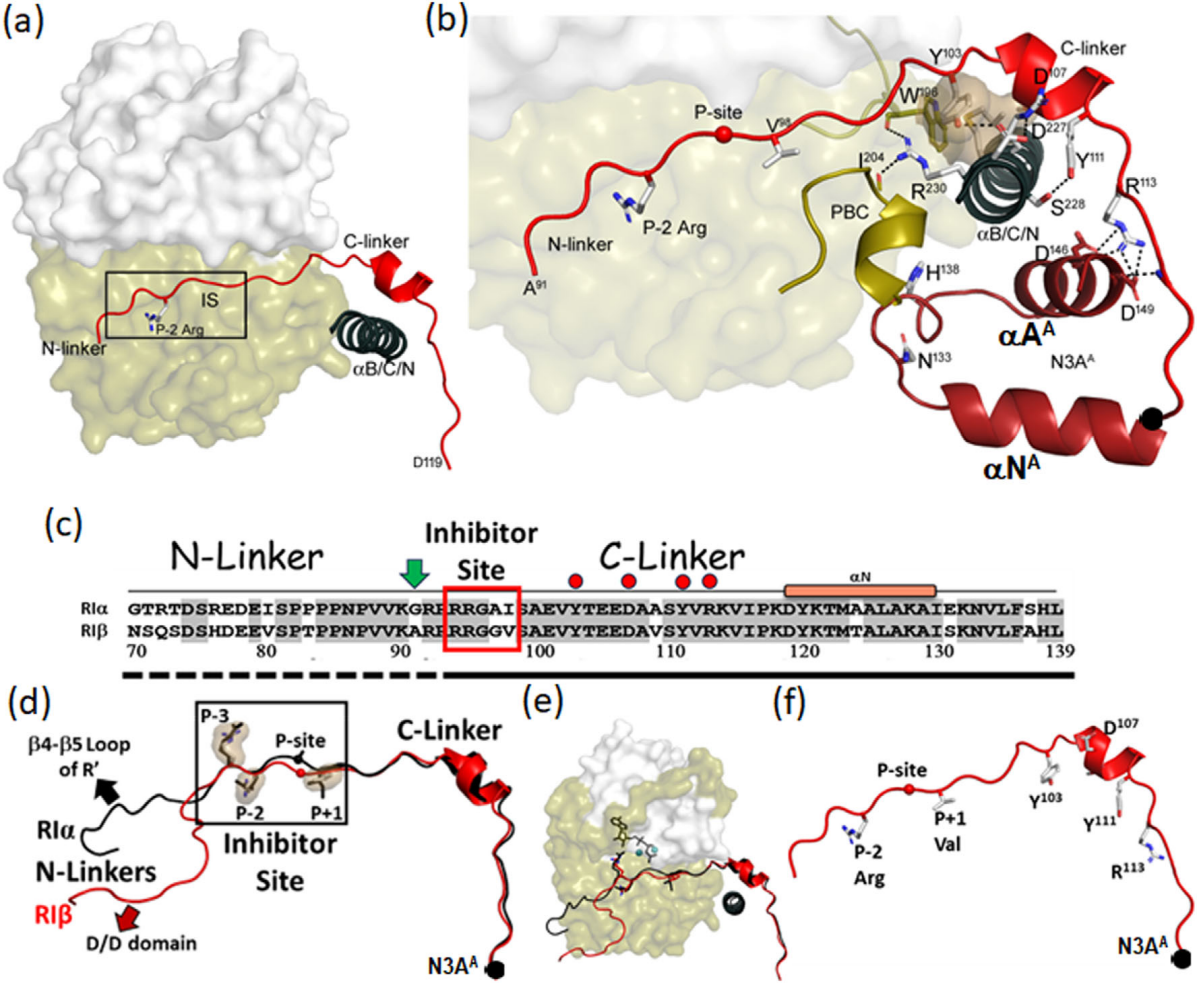
RIβ Inhibitor site docks into the active site cleft of C‐subunit. (a) The Inhibitor site (red) docks to the C‐subunit. The C‐subunit is shown as a surface representation, with N‐ and C‐lobes in white and tan, respectively. The P‐2 Arg and αB/C/N helix (dark blue) are also shown. (b) Zoom‐in view of C‐linker. The C‐linker wraps around the αB/C/N helix, some key residues from linker to N3A^A^ are highlighted. (c) Sequence alignment of the Inhibitor site. The green arrow marks the first residue, A91, in this RIβ:C structure. Four key C‐linker residues are labeled in red circles. (d) Structural alignment of Inhibitor sites. The N‐linkers have different conformations in RIα:C (purple) and RIβ:C (red), whereas the Inhibitor sites and C‐linkers are superimposed well. (e) The same view as (d) when docking into the C‐subunit. (f) The Inhibitor site of RIβ:C, some key residues are also shown.

The inhibitor site that resembles a PKA substrate is docked into the active site cleft of the C‐subunit (Figure 2a) where it bridges the N‐ and C‐lobes of the C‐subunit. The IS in RIα and RIβ is a pseudo‐substrate, similar to PKI, while in RII subunits the IS is a substrate where the Ser at the P‐site undergoes auto‐phosphorylation. In this RIβ structure, density is seen for the side chain of the P‐2 Arg; however, no density is seen for the P‐3 Arg side chain (Figure S4). Since this side chain typically interacts with the ribose of ATP, it is reasonable that it is disordered given that ATP is missing. In RIβ the P + 1 residue is a valine in contrast to isoleucine in both RIα and PKI, which both bind synergistically with high affinity when ATP(Mg)_2_ is present (Taylor et al. 2023). This synergistic high‐affinity binding that correlates with the closing of the active site cleft may be diminished in RIβ due to the replacement of Gly and Val at the P‐site and the P + 1 site compared to RIα (Figure S5).

#### 2.4.2 N‐terminal and C‐terminal linkers

The IS is flanked by the N‐linker and the C‐linker, and these two regions play different roles. While the N‐linker is fused directly to the D/D domain and is sensitive to nucleotide binding to the N‐lobe of the C‐subunit and possibly to the oxidation state of the D/D domain, the C‐linker is not only stably anchored to the C‐lobe of the C‐subunit, but also wraps around the helices of cAMP binding domain A (Figure 2). Comparing the linker sequences of RIα and RIβ (Figure 2) shows that the C‐linker is highly conserved. There is only one difference (A109V) in the C‐linker region that follows the IS. In contrast to the more variable N‐linker, which docks in different ways in the various R:C complexes, the structure of the entire C‐linker that follows the IS, including all of the side chains, is clearly seen and superimposable in all of the RIα and RIβ holoenzyme complexes, and B factors suggest that this region is quite stable (Brown et al. 2009; Ilouz et al. 2012; Kim et al. 2005; Kim et al. 2007; Wu et al. 2007). In the holoenzymes the C‐linker, which is disordered in the free R‐subunit, is specifically wrapped in a very stable way around the extended B/C helices of the CNB‐A domain, which are fused directly to the N‐Helix in the CNB‐B. All of the side chains in the C‐linker, including the short helix, are well‐resolved. Both hydrophobic (Y103, Y111) and charged (D107) residues in the highly conserved C‐linker interact with the B helix of CNB‐A while R113 caps the A helix of CNB‐A (Figure 2b). The C‐linker also interacts with the PBC of CNB‐A. Because the C‐linker directly controls access to both N3A motifs, it is a critical part of the allosteric switch that mediates activation of PKA by cAMP; it not only contributes to the stability of this region but also shields the active site of the C‐subunit from activation in the absence of cAMP. In addition to its anchoring to the C‐linker, the N3A motif in CNB‐A contributes to an RIβ:RIβ dimer interface in the RIβ:C complex.

### 2.5 Dimer interfaces differ in RIβ_2_C_2_ and RIβ:C

**FIGURE 3:**
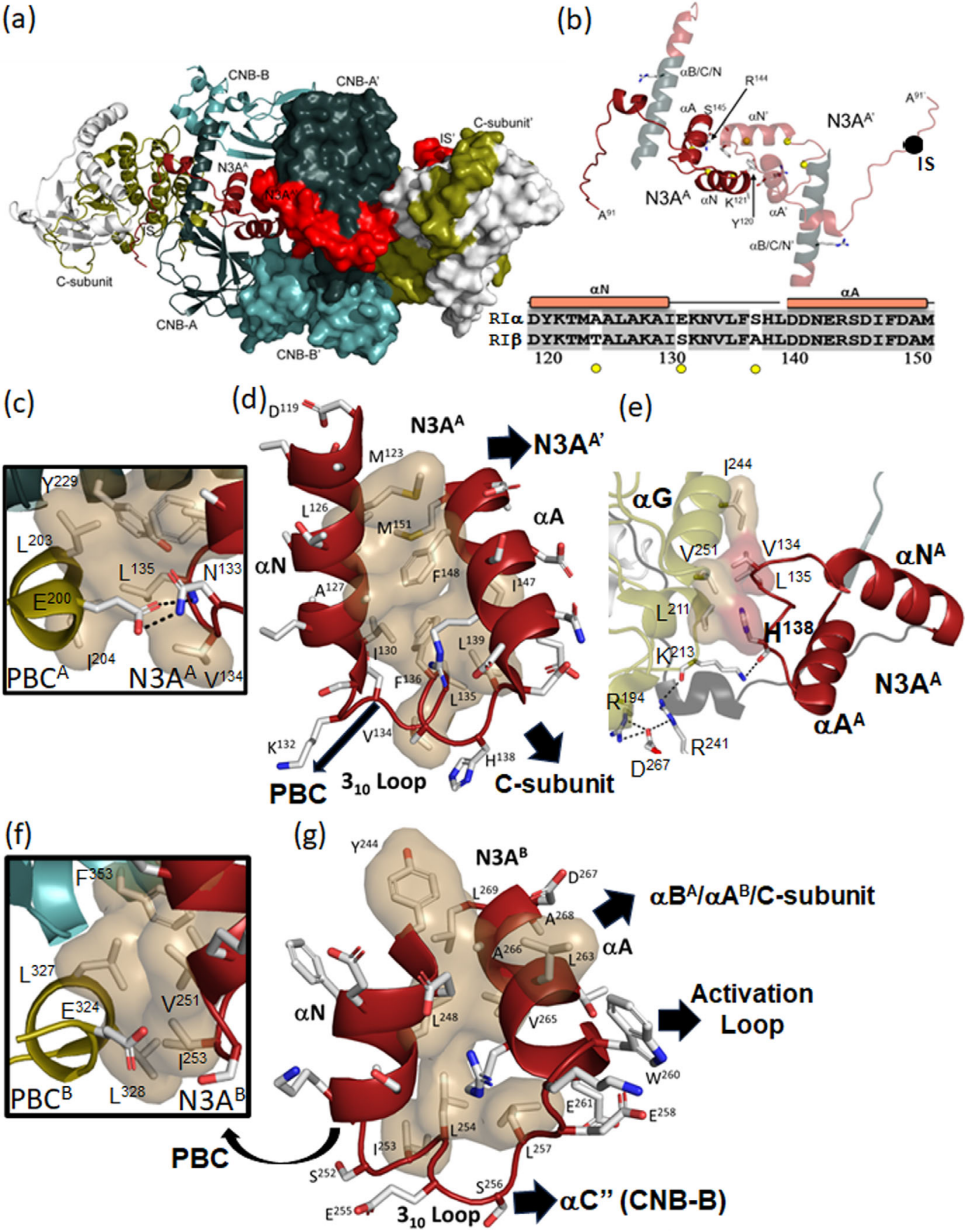
The N3A motifs nucleate multiple protein: protein interfaces and are stable rigid bodies. (a) Crystal packing of RIβ:C heterodimer structure. RIβ:C is shown in cartoon, and RIβ:C′ in surface representation. The color coding are the same as Figure 1a. (b) The N3A^A^:N3A^A^′ dimer interface. Four residues on the interface, Y120, K121, R144, and S145 are labeled. The sequence alignment of the N3A^A^ in RIα and RIβ are shown on the bottom. (c) The interface between N3A^A^ and PBC^A^. The hydrophobic shells are colored in sand, and H‐bonds are labeled with black lines. (d) N3A^A^ is a stable rigid body. The hydrophobic shell within N3A^A^ is colored in sand. The black arrows show the different domain interfaces of N3A^A^. (e) The interface between N3A^A^ and C‐subunit. V134 and H138 (red shell) hydrophobically pack to αG helix of C‐subunit (sand shell). (f) The interface between N3A^B^ and PBC^B^. (g) N3A^B^ is also a stable rigid body, the hydrophobic shells are in sand.

In contrast to the RIβ_2_C_2_ holoenzyme complex where ATP binding was stabilized by a head‐to‐head anti‐parallel packing of the C‐tail wrapped around the N‐lobe of the kinase domain, in the RIβ:C complex where ATP is missing, the N3A motif in the CNB‐A domain (N3A^A^), based on crystal packing, is the major dimer interface (Figure 3a,b). Although this dimer interface was observed in the packing of our original RIα monomer (Su et al. 1995), the importance and significance of this N3A:N3A′ dimer were only fully appreciated when a structure of the full‐length RIα dimer was solved (Bruystens et al. 2014). The N3A:N3A′ motif is also a dimer interface based on crystal packing in several of the RIα_2_C_2_ holoenzyme complexes that have been solved (Cao et al. 2019; Lu et al. 2019). In the absence of the C‐subunit (i.e., the cAMP‐bound R‐dimer) and in the R:C complex in the absence of ATP, this dimer interface appears to be dominant, although it is likely a low affinity interface that does not necessarily survive gel filtration. The biological importance of the N3A:N3A′ dimer in RIα is confirmed, however, by disease mutations at this interface. Carney Complex (CNC) mutations (R144S and S145G), for example, as well as by site‐specific mutations (Y120A and K121A), all make the RIα holoenzyme easier to activate in addition to altering the Hill Coefficient (Bruystens et al. 2014). In addition, small angle X‐ray scattering of the Y120A and K121A mutants confirmed global changes in conformation (Bruystens et al. 2014).

### 2.6 C‐terminal tail in the RIβ subunit is disordered in the RIβ:C complex

**FIGURE 4:**
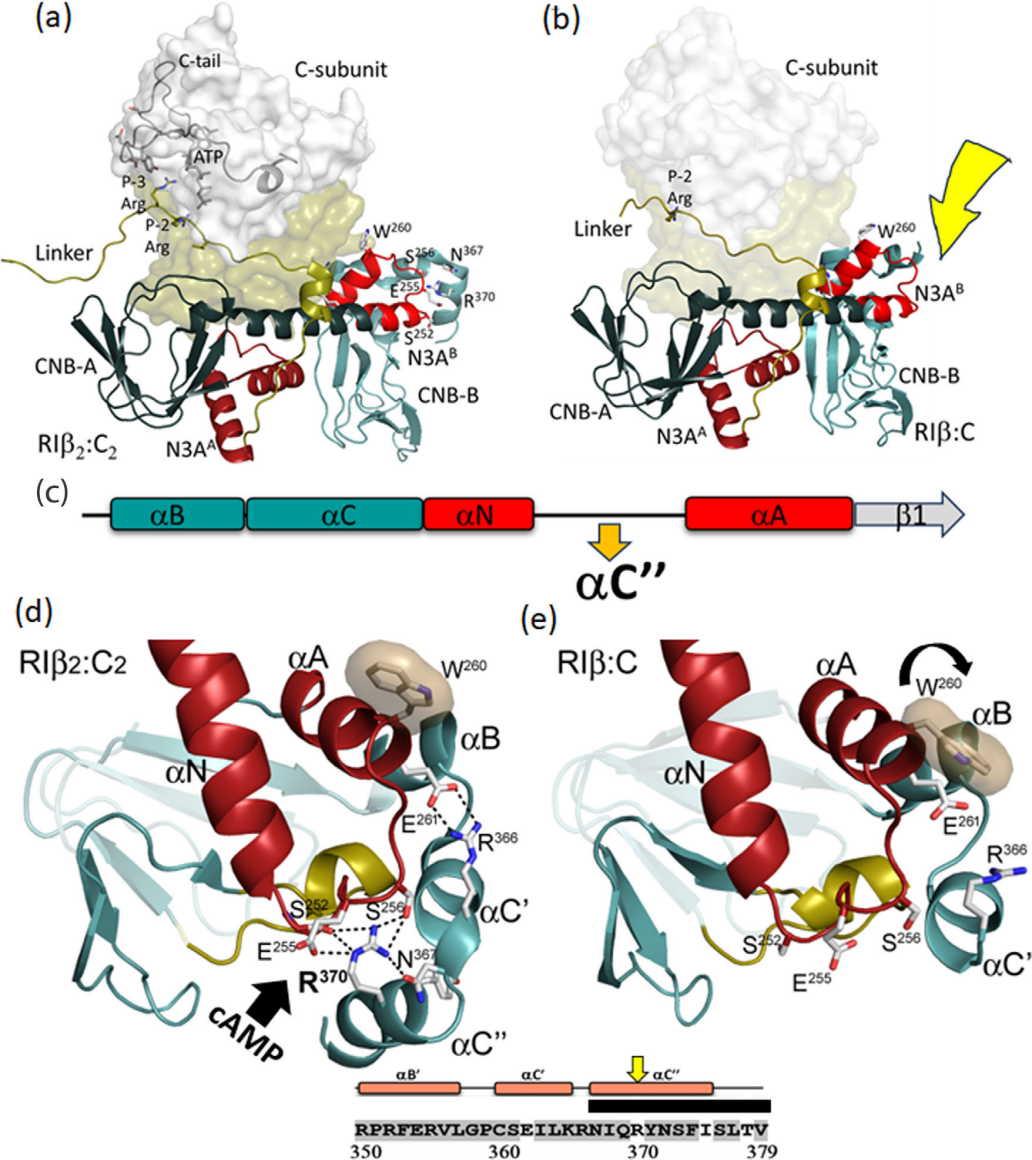
C‐terminal Tail in RIβ subunit is disordered. (a) The C‐tail of RIβ and ATP in C‐subunit are ordered in the structure of RIβ_2_C_2_ holoenzyme (PDB: 4DIN). C‐subunit is shown as surface representation with its C‐tail and ATP in black. The CNB‐A domain is colored in dark blue with N3A^A^ in dark red, and the CNB‐B domain in light blue with N3A^B^ in red, respectively. (b) RIβ:C heterodimer in the same view and color coding as (a). Residues 367–379 at the C‐terminus is not visible, marked by a yellow arrow. (c) Secondary structure elements near N3A^B^ are shown in cartoon. (d) Zoom‐in view of N3A^B^ motif in RIβ_2_C_2_ structure. R370 from C″‐helix forms several H‐bonds to 3_10_ loop of N3A^B^. PBC^B^ is colored in golden. W260 (sand shell), as well as E261 H‐bonding to R366 is also shown. (e) N3A^B^ motif in RIβ:C structure, the same view as (d).

A final striking feature of this RIβ:C structure lies in the CNB‐B domain. While the density for the B and C′ helices is clear, density for the C″ helix is completely missing suggesting that it is flexible (Figure 4b,e). In all our previous structures of RIα:C and RIα_2_C_2_ (Kim et al. 2007; Lu et al. 2019), as well as our previous structure of the RIβ_2_C_2_ holoenzyme (Ilouz et al. 2012), this density was clearly visible (Figure 4a,d). This helix is a major contributor to protein–protein interfaces, and most residues are highly conserved in RIα and RIβ (Figure S1). Another important interaction that is broken in the RIβ:C dimer involves R366 in the αC′ helix that immediately precedes the αC″ helix in CNB‐B. R366 forms a strong electrostatic interaction with E261 in the αA^B^ helix, and in all of our other structures, including the previous RIβ_2_C_2_ holoenzyme, this electrostatic interaction with E261 is conserved (Figure 4d). This interaction between E261 and R366 is also noteworthy because W260 in the A‐helix is the capping residue for cAMP bound to the CNB‐A domain. In the RIβ:C complex the side chain of R366 is seen, but it is more solvent‐exposed and no longer interacts with E261 (Figure 4e). In addition, the side chain of W260 has rotated significantly (Figure 4d,e).

Two key residues lie in the C″ helix. In the cAMP‐bound conformation (B‐form), the adenine rings of both cAMP molecules are capped by a hydrophobic residue (Figure S6c,d), and it is the aromatic ring of Y371 that caps the adenine ring of cAMP bound to CNB‐B (Figure S6d). This capping of cAMP^B^ by Y371 is conserved in every PKA R‐subunit. In the RIβ_2_C_2_ structure Tyr371 is solvent exposed while in the RIβ:C complex Y371 is unresolved (Table S2). The residue that is adjacent to Y371 is R370, and this residue also plays a major role by docking onto the N3A motif of CNB‐B. In RIα, R370 is replaced with N370, and this is one of the few sequence differences in the CNB domains of RIα and RIβ (Figures S1 and S7). To fully appreciate the importance of R370, one needs to understand the two N3A motifs that drive the allosteric communication that leads to inhibition and activation of the PKA C‐subunit. While we have previously characterized the N3A motif in CNB‐A (N3A^A^) as a dimer interface, which is also seen here in the RIβ:C complex (Figure 3a,b), the role of the N3A motif in CNB‐B (N3A^B^) has been less studied, and the biophysical features of this important motif (e.g., its conformational dynamics depending on cAMP binding) have only been recognized in single molecule optical tweezers experiments (Chau et al. 2023).

### 2.7 Structural and functional characterization of the N3A motifs

The N3A motif is a stable rigid body that contributes to multiple interfaces. The stability of the N3A motif is due largely to the tight and highly conserved hydrophobic packing of the amphipathic αN and αA helices, which leaves the exposed surfaces of the αN and αA helices, as well as the 3^10^ loop that joins the two helices, free to interact with other motifs including PBC residues (Figure 3d). The residues that form the hydrophobic core in N3A^A^ are shared by RIα and RIβ (Figures 3c and S7) and the strong hydrophobic packing of the two helices suggests that this motif will fold independently as a stable motif, probably as it comes off the ribosome. The 3^10^ helical segment that links the αN and αA helices is also anchored to the hydrophobic core through V134, L135, and F136 in N3A^A^ (Figure 3d). In contrast to the hydrophobic core of the N3A motifs, the outward‐facing residues contribute to many domain interfaces. The outward‐facing surface of the A^A^ helix, for example, is packed tightly against its own B^A^ helix so that the two ends of the 8‐stranded β‐sandwich are solidly anchored to each other. In contrast to the hydrophobic core residues that face inward, the outward‐facing residues of the N3A^A^ motif reach out to both the PBC^A^ and to the C‐subunit. In the holoenzyme complex, H138, as well as the side chain of V134, makes important surface contacts with the C‐lobe of the C‐subunit (Figure 3e) while N133 in the 3^10^ helix interacts with the highly conserved E200 in the PBC of N3A^A^.

In contrast to the N3A^A^ motif (Figure 3a–d), the importance of the N3A^B^ motif that flanks the two CNB domains has not been fully appreciated. Like the N3A^A^ motif, the N3A^B^ motif is highly conserved in RIα and RIβ (Figure S7). It is also very stable due to the hydrophobic packing of the two helices (Figure 3f,g) as well as V251 and I253 in the 3^10^ loop. Like the N3A^A^ motif, the hydrophobic core is a scaffold for displaying other residues that contribute to many interfaces as discussed below. It is also a key nucleation site for the highly dynamic protein–protein interfaces that allow for cross‐communication not only with the C‐subunit but also between the two CNB domains (Akimoto et al. 2015; Chau et al. 2023).

Some of the important hydrophobic interactions associated, in particular, with the 3^10^ loop in the N3A^B^ motif are conserved in the RIβ:C dimer while others are not. For example, V251 and I253 reach over to hydrophobic residues in the PBC^B^ motif such as L327 and L328 (Figure 3f), and this hydrophobic anchoring of the 3^10^ Loop to the PBC is conserved in both RIβ structures and in all RIα structures. It is also similar to CNB‐A (Figure 3c). In contrast, a key functional residue in the C″ helix, R370, is missing in the RIβ:C dimer. In the RIβ_2_C_2_ holoenzyme, R370 interacts with several residues in the 3^10^ Helix of N3A^B^ (Figure 4d). In the RIβ:C dimer, capping of the 3^10^ Loop by R370 is abolished in contrast to the hydrophobic interactions with the PBC residues. Although the sequences in the CNB domains are overall highly conserved in RIα and RIβ, R370 is one exception. In RIα Arg370 is replaced with Q370, so the capping of the 3^10^ helix is significantly different and likely much weaker in RIα (Figure S8).

The other key functional residue in the C″ helix is Y371. In the cAMP‐bound conformation (B‐form), the adenine rings of both cAMP molecules are capped by a hydrophobic residue (Figure S6c,d), and it is the aromatic ring of Y371 that caps the adenine ring of cAMP bound to CNB‐B (Figure S6d). This capping of cAMP^B^ by Y371 is conserved in every PKA R‐subunit. In contrast to R370, the side chain of Y371 is exposed to solvent in the RIβ_2_C_2_ holoenzyme, while both residues are solvent‐exposed in RIβ:C (Table S2).

Surprisingly, both capping residues are located in CNB‐B. In both RI subunits W260 caps cAMP bound to CNB‐A (Figures S6c and 6d). In both RI:C complexes, however, W260 is located at the R:C interface; specifically, it is packed against K192 in the Activation Loop of the C‐subunit (Akimoto et al. 2015; Chau et al. 2023; Ilouz et al. 2012). Thus, unlike R333 and Y371, W260 plays an important functional role in both the holoenzyme and in the free R‐subunit. In the RIβ:C structure, W260 is still at the R/C interface, but the indole ring has rotated (Figure 4d,e). Observing different conformations for W260 in the RIβ:C structure is a final discovery that comes from our comparison of the two structures. This is consistent with the importance of W260 dynamics described previously for RIα:C complexes (Akimoto et al. 2015; Chau et al. 2023).

### 2.8 Allosteric hotspots in the RIβ/RIα holoenzymes

**FIGURE 5:**
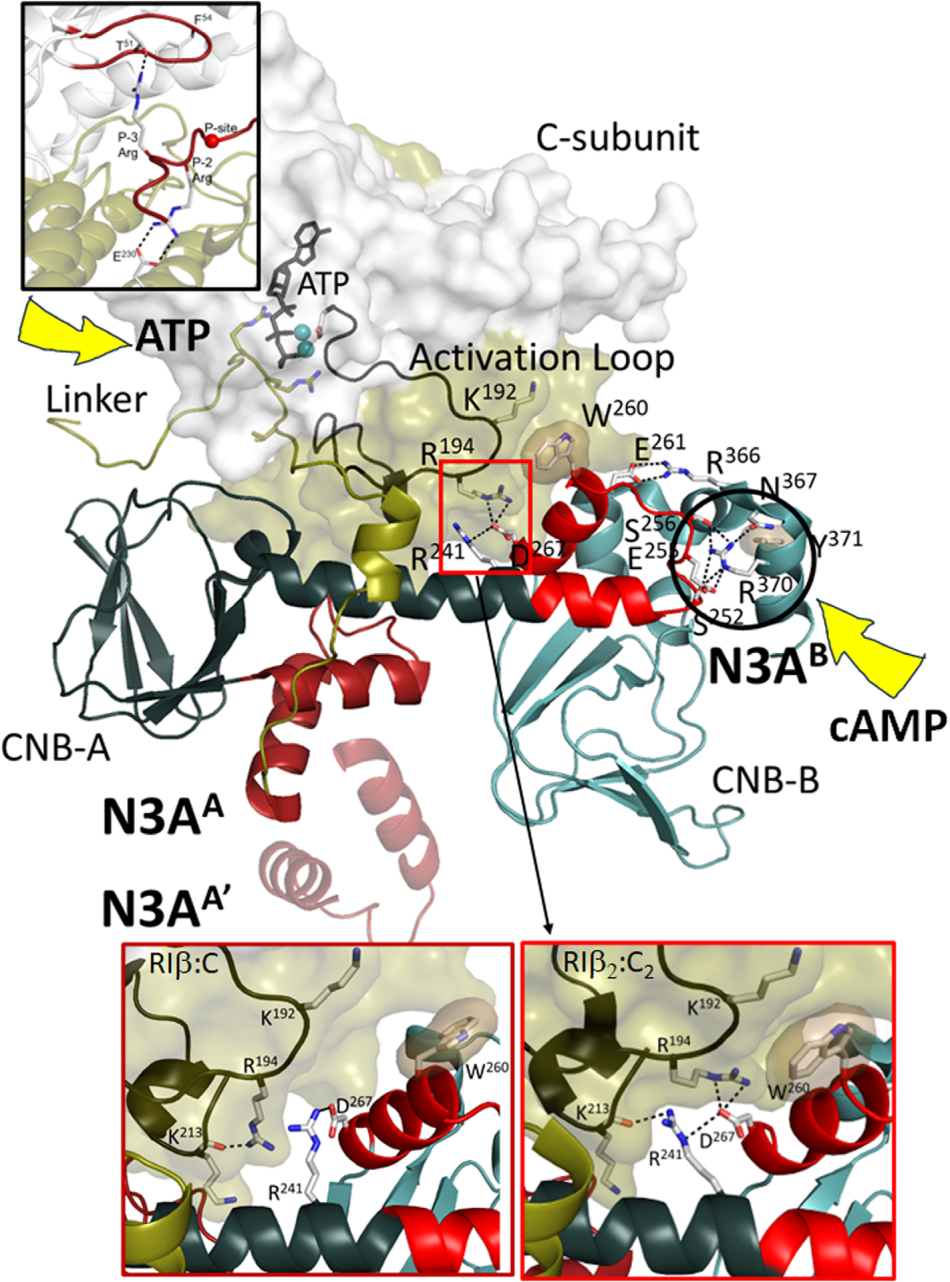
Different functional roles of N3A motifs. N3A^A^ motif (dark red) serves as a dimer interface. N3A^B^ motif (red) is on the R:C interface with the Activation loop of C‐subunit. The yellow arrows show the binding sites of cAMP and ATP (black) in RIβ_2_C_2_ structure. Top inset shows the ATP is missing from the RIβ:C structure. Bottom inset (right) shows the R:C interface of N3A^B^ in RIβ_2_C_2_, whereas (left) shows the same interface in RIβ:C structure. W260 (sand shell) has a side‐chain flip, and R194‐D267 interactions were broken.

Figure 5 highlights not only the two N3A motifs and the extended B/C/N helix, a characteristic feature of all PKA holoenzymes, but also a second major allosteric node at the R/C interface. D267 at the C‐terminus of the αA helix in CNB‐B (αA^B^) is part of a conserved allosteric node at the R:C interface where it interacts directly with R241 in the αB helix of CNB‐A and R194 in the activation loop of the C‐subunit in RIβ_2_C_2_. This allosteric node is a major hub for R:C communication in both full‐length RIα and RIβ holoenzymes, but in the RIβ:C complex, the interface with R194 is changed (bottom insets, Figure 5). The side chain of R194, in particular, no longer interacts with D267, whereas the interactions between R241 and D267 are conserved as they are in all states, even in the dissociated cAMP‐bound RI‐subunits (Su et al. 1995). These interactions are also conserved in the RII subunits. At the other end of the αA^B^ helix is W260, which will become the capping residue for cAMP bound to CNB‐A. In addition to being close to the D241/D267 allosteric node, W260 is also close to the activation loop of the C‐subunit. In the RIβ_2_C_2_ holoenzyme ATP is stably bound to the active site cleft of the C‐subunit, while in the RIβ:C complex, ATP is missing, and the cleft is open. In this RIβ:C complex, where ATP is missing and the C″ helix is disordered, the conformation of the indole side chain of W260 rotates (Figures 4 and 5). We hypothesize that the αA^B^ helix, flanked by two sensitive motifs (D267 and W260), transmits the information of cAMP binding to the C‐terminal PBC in CNB‐B at the R/C interface (Figure 5). The increased mobility of the indole side chain of W260 makes it poised for binding cAMP to CNB‐A, which will eventually lead to a major conformational reorganization of the CNB‐B domain that allows it to interact directly with cAMP^A^ instead of with the C‐subunit.

## 3 DISCUSSION

Unlike the RII subunits, which have very stable D/D domains that spontaneously form a four‐helix bundle and are always anchored through AKAPs to membranes, both RI subunits have D/D domains that are redox‐sensitive due to two interchain disulfide bonds (Banky et al. 2000; Brennan et al. 2006; Bubis et al. 1987; Sarma et al. 2010). The four‐helix bundle is stable when the disulfide bonds are formed, but unstable when the cysteines (Cys16 and Cys37) are reduced (Banky et al. 2000; Kinderman et al. 2006). The RIα D/D domain can also be stabilized by binding to AKAPs (Banky et al. 2000; Bubis et al. 1987). Although when RIα is expressed in *Escherichia coli*, the disulfide bonds are mostly oxidized (Bubis et al. 1987). In cardiac myocytes, in the absence of redox stimulation, the disulfide bonds in RIα appear to be mostly reduced (Haushalter et al. 2019). In RIα purified from porcine muscle, the two chains are disulfide bonded (First and Taylor 1984). In our original structure of the RIβ_2_C_2_ holoenzyme, the D/D domain was reduced and partially unfolded (Ilouz et al. 2012) while in human brain, the RIβ subunit appears to be disulfide bonded (Figure S2) (Benjamin‐Zukerman et al. 2024). We thus asked what would happen when the crystal was oxidized by diamide or bound to an AKAP peptide, which would also stabilize the D/D domain (Kinderman et al. 2006; Sarma et al. 2010). Both conditions caused the crystals to become unstable and suggested that nucleotide was lost. To capture this at higher resolution we thus co‐crystallized the RIβ:C holoenzyme in the presence of an AKAP peptide. The resulting structure was a monomer of RIβ that had been cleaved in the N‐linker region in complex with the C‐subunit. This fragment was confirmed by gel electrophoresis of the crystal (Figure S3d). Although some proteolysis was observed in previous purifications of the RIβ subunit and during the storage of the free RIβ subunit, no significant cleavage was observed during storage once the holoenzyme was formed (Figures S3c and S9). Mass spec analysis of the spontaneous cleavage product confirmed that the D/D domain was missing (Figure S10). While it is still important to correlate the cleavage of RIβ with the addition of the AKAP and to elucidate the relationship between the oxidation state of the D/D domain and the R:C domains, in this study we compare the structure of the RIβ:C complex with the structure of the RIβ_2_C_2_ complex. While the linker is disordered in all free R‐subunits, we show how in the ATP‐bound RIβ_2_C_2_ complex the N‐linker senses ATP while the C‐linker folds over not only the N3A^A^ motif, but also over the N3A motif in CNB‐B (N3A^B^) and blocks access of cAMP. In contrast, in the RIβ:C complex, ATP is missing and interactions of the C‐terminal Helix with the N3A^B^ motif are abolished. The structure, function, and dynamic features of the linker and of the two highly conserved and stable N3A motifs in the RIβ:C complex are compared to their role in the RIβ_2_C_2_ complex. Finally, we show how the side chain of W260 in the αA^B^ Helix rotates in the RIβ:C dimer suggesting that it may be a dynamic sensor for cAMP mediated activation of the holoenzyme.

Our analysis of this RIβ:C structure clearly distinguishes three interaction regions of the linker (Figure 2). The inhibitor site in the holoenzyme docks into the active site cleft, and for high‐affinity binding, this requires ATP for pseudo‐substrate inhibitors like RI subunits and PKI. The N‐linker contributes to this high‐affinity binding of ATP, and, in the case of the previous RIβ_2_C_2_ holoenzyme complex, this site, which includes the FDDY motif in the C‐tail that binds to the adenine ring, was further stabilized by an anti‐parallel dimer packing interface where the C‐tail of one R:C protomer was packed against the C‐tail of the adjacent R′:C′ protomer (Figure S1). The C‐linker, in contrast, is anchored in a very stable and conserved way onto the CNB‐A domain. It specifically wraps over the N3A motif, the PBC, and the B/C/N helix, positioning the CNB‐A domain in close proximity to the C‐lobe of the C‐subunit. This region includes the interface with the Activation Loop of the C‐subunit and is the site that needs to be disrupted when cAMP is bound. The C‐linker is thus committed to shielding the C‐lobe from activation by cAMP, in contrast to the N‐linker, which regulates ATP binding to the N‐lobe.

While the cAMP binding sites in the free R‐subunits and in the various holoenzyme complexes have been extensively studied, the N3A motifs have not been systematically analyzed and compared previously. Each CNB domain consists of a β‐sandwich immunoglobulin‐like fold that contains eight β strands and helical motifs flank both ends of the β sandwich (Berman et al. 2005; Kannan et al. 2007; McKay and Steitz 1981; Su et al. 1995). Embedded between β6 and β7 is a third small helix, referred to as the Phosphate Binding Cassette (PBC), which is the docking site for cAMP and the signature motif for every CNB subunit (Berman et al. 2005). The naming of the CNB helices in RIα (Su et al. 1995) was based originally on the structure of the Catabolite Gene Activator Protein (CAP), which had been solved previously by Steitz (McKay and Steitz 1981). This was the first and only previous structure that described a CNB domain (Berman et al. 2005), and in CAP there is no N‐helix. The N‐helix appears to have evolved as a stable and conserved part of the CNB domain in bacteria, perhaps when another domain or protein is fused to the CNB domain (Kannan et al. 2007), but obviously the origin of the N helix as a stable part of most CNB domains needs to be further explored. Importantly, however, the N3A motif is highly conserved in both CNB domains of all PKA R‐subunits and in the cGMP binding domains of all cGMP‐dependent protein kinases (Kannan et al. 2007).

Here we show how each N3A motif is very stable allowing it to function as a rigid body that we hypothesize will fold into a very stable motif independent of the rest of the polypeptide chain due to its highly conserved hydrophobic core (Figure 3). The N3A motif is a stable part of both CNB domains, and each N3A motif mediates essential interfaces between the two CNB domains and the protein–protein interface between the R and C subunits and between the R dimers. The outward‐facing surfaces of the N3A motifs provide multiple interaction sites that stabilize either the holoenzyme conformation (H‐form) or the cAMP‐bound conformation (B‐form) (Figure S6) (Akimoto et al. 2015; Hao et al. 2019). However, the N3A motif remains as a rigid body in both conformations. In this RIβ:C conformation, the N3A^A^ motif also serves as a dimer interface (Figure 3), which is different from the FDDY interface that we saw in the RIβ_2_C_2_ holoenzyme, which was stabilized by the head‐to‐head dimer of the two N‐lobes (Ilouz et al. 2012) (Figure S1). Appreciating the stability of the N3A domain as a multi‐valent interaction motif that mediates allosteric cross talk between the two CNB domains and the C‐subunit is a major finding of this present work.

**FIGURE 6:**
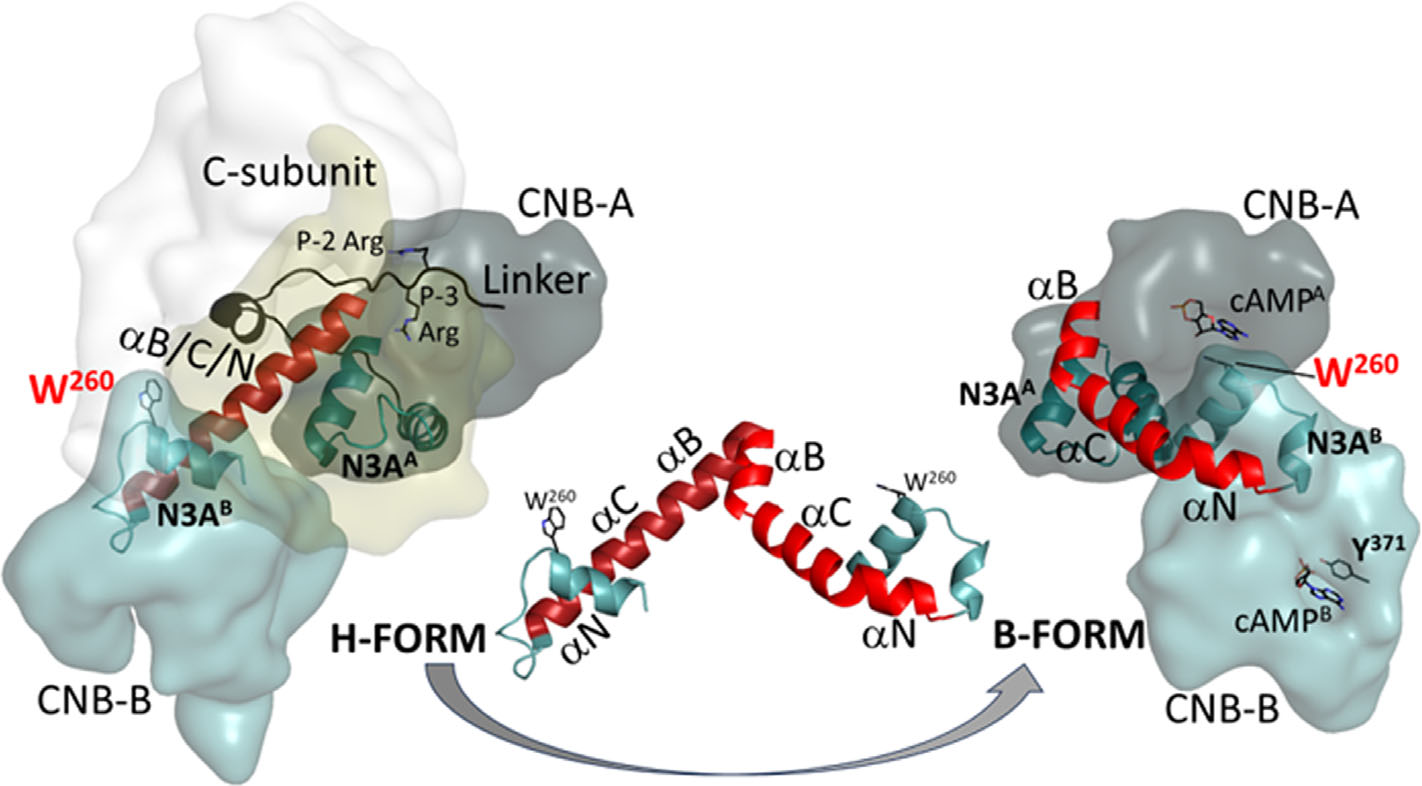
The extended BCN helix joining the two CNB‐domains. (left) The RIβ:C structure shown in surface representation (H‐form). The N‐ and C‐lobe of C‐subunit are shown in white and tan, the CNB‐A and CNB‐B domains of RIβ are in dark blue and light blue, respectively. The extended BCN helix is in red. (right) The cAMP bound RIα structure shown in surface representation (B‐form). (middle) The structural comparison of BCN helices in two states.

To understand how the stepwise activation of the PKA holoenzyme is achieved, it is essential to understand how the two N3A motifs help to control the BCN helix that directly links CNB‐A to CNB‐B. In the holoenzymes, this helix is extended in a single long helix and in RI subunits. W260 in the αA helix that is part of the N3A^B^ motif is docked to the activation loop of the C‐subunit (Figure 6). In contrast, the B/C helices at the end of CNB‐A are shielded by the N3A^A^ motif and by the C‐linker that is stably docked onto the C‐lobe of the C‐subunit. In the fully dissociated RI subunit, the BCN helix is bent at two key sites, which allows for the CNB‐B domain to undergo a conformational change that positions W260 to cap cAMP bound to CNB‐A (Berman et al. 2005; Su et al. 1995). While the detailed steps of this transition will require much work at many scales, we highlight here the critical role of W260 as a dynamic sensor that helps to mediate this transition.

Our model suggests that the binding of cAMP to the C″ helix of CNB‐B is sensed by W260 at the R/C interface, and that this is likely the initial event that leads to the binding of the second molecule of cAMP to CNB‐A. This ordered binding of cAMP first to CNB‐B and then to CNB‐A has been computationally modeled previously for RIα (Boras et al. 2014). Our comparison of RIβ_2_C_2_ to RIβ:C suggests that the removal of ATP can also help to “prime” W260 for recognition of the second cAMP. Binding of ATP and two Mg ions locks the C‐subunit into a fully closed conformation when the C‐subunit is bound to a pseudo‐substrate such as the heat stable protein kinase inhibitor or the RI subunits (Kim et al. 2007; Zheng et al. 1993). This leads to synergistic high‐affinity binding of both ATP (60 nM) and PKI/RIα (sub nM) (Herberg et al. 1999). The slow endogenous hydrolysis of ATP by the C‐subunit will abolish this high affinity binding and lead to the opening of the active site cleft. We propose here that this change at the cleft interface can be sensed by W260 in both RIβ and RIα. While many questions remain to be answered both at the structure level and at the computational level, our comparison of these two RIβ structures provides new hypotheses to be tested. These will be important studies because many disease mutations lie in both RIα and RIβ. These insights may also provide a molecular foundation for future studies aimed at dissecting the pathophysiological consequences of RIβ mutations—and may ultimately enable targeted therapeutic strategies for RIβ‐related neurological disorders.

## 4 MATERIALS AND METHODS

### 4.1 Purification of RIβ from tissues

The tissues from different mouse organs (brain, heart, liver, and spleen) were homogenized using lysis buffer (150 mM NaCl, 50 mM HEPES pH 7.4, 25 μg/mL Digitonin, protease and phosphatase inhibitors at 1:200 dilution) in volumes adjusted according to the weight of each organ, using a standard of 10 μL of buffer per 1 mg of tissue. Organs were homogenized on ice, and the suspension was incubated with end‐over‐end rotation for 10 min at 4°C. After incubation, the samples were centrifuged at 2000 RCF for 10 min at 4°C. The supernatants were collected for Western blot analysis.

### 4.2 Disulfide bonding classification using SDS‐PAGE and Western blot

Protein concentrations were determined using the Bradford assay in a plate reader (i‐CONTROL). Each protein sample (70 μg) was mixed with 5× sample buffer with or without a reducing agent. Reduced samples were heated at 95°C for 4 min before loading, while non‐reducing samples were left unboiled. Proteins were separated by 8% SDS‐PAGE. After electrophoresis, proteins were transferred onto PVDF membranes (Bio‐Rad). The membranes were then blocked with 5% bovine serum albumin (BSA) (Sigma) in 1× TBST for 1 h at room temperature. Primary antibody incubation occurred overnight at 4°C, followed by four washes with TBST. Membranes were then incubated with HRP‐conjugated secondary antibodies for 1 h at room temperature, washed four more times with TBST, and incubated for 1 min with an ECL reagent. Signal detection was performed using the UVITEC CAMBRIDGE enhanced chemiluminescence system.

### 4.3 Protein expression and purification of bovine wild‐type full‐length C‐subunit

Bovine wild‐type full‐length C‐subunit was expressed and purified as previously described (Cànaves et al. 2000). The C‐subunit, containing three phosphorylation sites (S10, T197, and S338), was isolated from Peak II and used for crystallography. Human wild‐type full‐length RIβ was expressed in *E. coli* BL21 (DE3) using overnight media (Sigma) at 37°C. The protein was purified from inclusion bodies as previously described (Ilouz et al. 2012). Throughout all purification steps, the following protease inhibitors were used: 10 mM benzamidine, 0.4 mM AEBSF, 1 μM Pepstatin, 1 μM Leupeptin, 28 μM TPCK, and 28 μM TLCK.

### 4.4 Formation of the holoenzyme complex

Wild‐type full‐length RIβ subunit was combined with wild‐type C‐subunit phosphorylated at Ser10, Ser197, and Ser338 in a molar ratio of 1:1.3. This mixture was subjected to dialysis against a buffer solution containing 10 mM MES (pH 6.5), 5 mM to a final concentration of 12 mg/mL. The renatured RIβ protein demonstrated proper function by binding to cAMP‐Sepharose resin and interacting appropriately with the C‐subunit, MgCl2, 50 mM NaCl, 0.5 mM ATP, and 5 mM DTT at 4°C overnight. Subsequently, excess C‐subunit was separated from the holoenzyme via gel filtration chromatography using a Superdex 200 column. The distinct peak, indicative of the pure holoenzyme complex, was collected and concentrated, confirming the recovery of its native three‐dimensional structure.

### 4.5 Soaking diamide, peptide, and cAMP into the crystals

To investigate the effect of disulfide bond oxidation on the D/D domain, we initiated soaking experiments with diamide at concentrations ranging from 1 mM to 10 mM. Subsequently, we co‐crystallized the RIβ holoenzyme with smAKAP peptide (TVILEYAHRLSQDILCDALQQWAC) at equimolar quantities and incubated the mixture at room temperature for 30 min. The resulting complex, at a final concentration of 12 mg/mL, was utilized for crystallization. After crystallizing the RIβ holoenzyme, we sought to introduce cAMP into the crystal drops at concentrations ranging from 0.1 to 10 mM. Our aim was to capture the intermediate state between the intact RIb2:C2 holoenzyme and its dissociated form. However, we encountered challenges during this process. At higher concentrations, the crystals dissolved, while at lower concentrations, no diffraction data were collected.

### 4.6 Crystallization conditions

The RIβ_2_:C_2_:(Mg2ATP)_2_ complex was co‐crystallized with the smAKAP peptide (sequence: TVILEYAHRLSQDILCDALQQWAC) at a molar ratio of 2:1. The peptide was initially dissolved in 0.1M Tris buffer at pH 8.5. Crystallization was achieved using the vapor diffusion method with the hanging drop technique. The drops contained 1.4M ammonium sulfate and 0.1M sodium acetate at pH 5.3. To control the evaporation rate, the reservoir was covered with a 50% silicon oil and 50% paraffin oil mixture. Crystals grew over 2 weeks to their maximum size, after which they were transferred to a cryoprotectant solution (mother liquor with 20% glycerol) and flash‐cooled in liquid nitrogen.

### 4.7 Data collection and processing

A complete dataset was collected from a single crystal at the ALS (Advanced Light Source) beamline 8.2.2. The crystal diffracted to a resolution of 3.7 Å and was indexed to the hexagonal space group P6522, with unit cell dimensions *a* = 103.5 Å and *c* = 313.4 Å. The asymmetric unit contained one molecule. Data were processed and scaled using the HKL2000 suite (Otwinowski and Minor 1997).

### 4.8 Structure solution and refinement

Initial phases for the quaternary complex were determined by molecular replacement, using RIα(91–379):C (PDB ID: 2QCS) and the isolated dimerization and docking domain (PDB ID: 3IM3) as search models. The asymmetric unit included a single heterodimer composed of one R subunit and one C subunit. Refinement was performed using Phenix (Liebschner et al. 2019), with 5% of reflections reserved for cross‐validation. Manual model building was conducted in Coot based on electron density maps. The final model encompassed residues 14–350 of the C subunit and residues 91–366 of the RIβ subunit. Validation through PROCHECK indicated good geometry, with no residues in disallowed regions of the Ramachandran plot.

### 4.9 Visualization

All structural figures were generated using Pymol.

### 4.10 Mass spectrometry

An old sample of previously purified RIβ was run on an SDS gel. A major band, at 38 kDa molecular weight, was cut and sent to the Bimolecular Mass Spectrometry facility, UCSD for proteomics analysis.

## 5 ACCESSION NUMBER

The atomic coordinates and structure factors have been deposited in the Protein Data Bank, www.pdb.org (PDB ID code 9O7V).

## AUTHOR CONTRIBUTIONS

**Jian Wu:** Investigation; validation; software; data curation; writing – review and editing. **Jessica G. H. Bruystens:** Writing – review and editing. **Puspashree Sahoo:** Investigation; methodology; formal analysis; data curation. **José Bubis:** Methodology; formal analysis. **Rodrigo A. Maillard:** Writing – review and editing; funding acquisition. **Susan S. Taylor:** Conceptualization; investigation; funding acquisition; writing – review and editing; supervision; validation. **Ronit Ilouz:** Conceptualization; investigation; funding acquisition; writing – original draft; methodology; validation; visualization; writing – review and editing; project administration; supervision.

## Supporting information


**Figure S1.** Crystal structure of RIβ_2_C_2_ holoenzyme. (top left) The structure of RIβ_2_C_2_ holoenzyme with the D/D domain visible (black circle). (top right) The anti‐parallel dimer interface between the C‐tails of the two C‐subunits. (bottom) Sequence alignment of human RIα and RIβ. The secondary structure elements are shown in cartoon.

**Figure S2.** Protein expression and dimerization of RIβ in mouse tissues. Western blot analysis of protein samples from brain, heart, liver, and spleen of mice were performed under reduced and non‐reduced conditions. (left, reduced conditions): Protein expression of RIβ was performed in reduced conditions, showing the monomeric form of the protein (~50 kDa) in all tissues, along with PKA catalytic subunit Cα. (right, non‐reduced conditions): RIβ is dimer in non‐reduced conditions, showing the dimeric form (~100 kDa) in brain. The blot was probed with the RIβ specific antibody.

**Figure S3.** Gel electrophoresis of the crystal confirmed cleavage of the full‐length RIβ. (a) The full‐length RIβ_2_C_2_ holoenzyme used for crystallization, as shown previously in Figure S2 (Ilouz et al. 2012). (b) The diffracting crystals contain the full‐length RIβ_2_C_2_ holoenzyme, as was also shown previously in Figure S2 (Ilouz et al. 2012). (c) Following purification of the RIβ_2_C_2_ holoenzyme, no degradation products were observed even after storage of the holoenzyme for 2 weeks at 4°C. (d) The diffracting crystal shows the RIβ had been degraded to a 38 kDa fragment, as shown by the red arrow and square. Panels (a, c) are SDS gels; panels (b, d) are silver stained gels.

**Figure S4.** Density maps of motifs in the kinase domain. (a) Top view of the G‐loop, 2Fo‐Fc density at 1σ are in gray. (b) Side view of the G‐loop and DFG motif, ATP (black) is from 1ATP when superimposed with the C‐subunit of RIβ:C structure. There is no density for ATP. (c) C‐tail of C‐subunit. The side chains of some key residues are also shown. (d) The Inhibitor site of RIβ and the Activation loop of C‐subunit. The P‐2 Arg interacts with E230, their density are well traced. (e) The Inhibitor site and FDDY motif. P‐3 Arg is flexible as its side‐chain density is not traced.

**Figure S5.** Inhibitor sites are different in RIα and RIβ. (left) Inhibitor site of RIβ docks into the active site cleft of C‐subunit. ATP is missing. (right) Inhibitor site of RIα docks into the active site cleft of C‐subunit. ATP is in black stick. (inset) The structural comparison of two Inhibitor sites. (bottom) The sequence alignment of the Inhibitor sites of four R‐subunit isoforms.

**Figure S6.** W260 plays important functional role. (a) N3A^B^ motif in RIβ_2_C_2_ structure. The hydrophobic surface among N3A^B^ (red), PBC^B^ (yellow) and C‐tail (light blue) are shown as white shell. W260 is circled in red. (b) N3A^B^ motif in RIβ:C structure, the same view as (a). N3A^B^ sequence alignment in RIα and RIβ is also shown. (c) W260 is the cAMP capping residue of CNB‐A in the cAMP bound RIα. The N3A^A^ is colored in red and N3A^B^ in dark red. (d) Y371, from the C″‐helix, is the cAMP capping residue of CNB‐B in the cAMP bound RIα. W260 and cAMP in CNB‐A are also shown.

**Figure S7.** The sequence alignment of CNB‐A (top) and CNB‐B (bottom) in RIα and RIβ. The secondary structure elements are shown in cartoon.

**Figure S8.** R370 is a key functional residue in RIβ. (left) R370 interacts with several residues in the 3^10^ loop of N3A^B^. (right) Q370 has much weaker interaction with N3A^B^ in RIα.

**Figure S9.** Purification of the RIβ through a gel filtration column. (left) The protocol for purification of RIβ. (right) Panel (a) shows the purification of the cGMP‐eluted RIβ. As seen in the gel (top right), there are some degradation products. Panel (b) shows the RIβ holoenzyme isolated on the same gel filtration column. As seen in the gels (bottom right), no breakdown products were observed. This holoenzyme was routinely pooled and stored at 4°C for many weeks; no degradation was observed (Figure S3c).

**Figure S10.** Mass spectrometry analysis of a 38 kDa cleavage product. The 38 kDa band from a stored sample of RIβ, one of the most abundant protein species in the analysis, was excised from a SDS gel and analyzed by mass spectrometry. The results confirm this sample was a degradation of the full‐length protein with most of the N‐terminus missing. The red arrow shows the first traceable residue in the RIβ:C structure.

**Table S1.** Data collection and refinement statistics.

**Table S2.** Residue contacts in RIβ in the Holo (H) and cAMP bound (B) conformation.

## Data Availability

The data that support the findings of this study are openly available in pdb_00002qcs at https://doi.org/10.2210/pdb2QCS/pdb, reference number 2QCS.

## References

[pro70332-bib-0001] Akimoto M , McNicholl ET , Ramkissoon A , Moleschi K , Taylor SS , Melacini G . Mapping the free energy landscape of PKA inhibition and activation: a double‐conformational selection model for the tandem cAMP‐binding domains of PKA RIα. PLoS Biol. 2015;13:e1002305. 10.1371/journal.pbio.1002305 26618408 PMC4664472

[pro70332-bib-0002] Arencibia JM , Pastor‐Flores D , Bauer AF , Schulze JO , Biondi RM . AGC protein kinases: from structural mechanism of regulation to allosteric drug development for the treatment of human diseases. Biochim Biophys Acta. 2013;1834:1302–1321. 10.1016/j.bbapap.2013.03.010 23524293

[pro70332-bib-0003] Bachmann VA , Mayrhofer JE , Ilouz R , Tschaikner P , Raffeiner P , Röck R , et al. Gpr161 anchoring of PKA consolidates GPCR and cAMP signaling. Proc Natl Acad Sci U S A. 2016;113:7786–7791. 10.1073/pnas.1608061113 27357676 PMC4948347

[pro70332-bib-0004] Banky P , Newlon MG , Roy M , Garrod S , Taylor SS , Jennings PA . Isoform‐specific differences between the type Ialpha and IIalpha cyclic AMP‐dependent protein kinase anchoring domains revealed by solution NMR. J Biol Chem. 2000;275:35146–35152. 10.1074/jbc.M003961200 10899163

[pro70332-bib-0005] Benjamin‐Zukerman T , Pane V , Safadi‐Safa R , Solomon M , Lev‐Ram V , Aboraya M , et al. Allosteric modulation of protein kinase A in individuals affected by NLPD‐PKA, a neurodegenerative disease in which the RIβ‐L50R variant is expressed. FEBS J. 2025;292(18):4808–4832. 10.1111/febs.70098 40244081 PMC12443466

[pro70332-bib-0006] Benjamin‐Zukerman T , Shimon G , Gaine ME , Dakwar A , Peled N , Aboraya M , et al. A mutation in the PRKAR1B gene drives pathological mechanisms of neurodegeneration across species. Brain. 2024;147(11):3890–3905. 10.1093/brain/awae154 38743596 PMC11531844

[pro70332-bib-0007] Berman HM , Ten Eyck LF , Goodsell DS , Haste NM , Kornev A , Taylor SS . The cAMP binding domain: an ancient signaling module. Proc Natl Acad Sci U S A. 2005;102:45–50. 10.1073/pnas.0408579102 15618393 PMC544069

[pro70332-bib-0008] Boettcher AJ , Wu J , Kim C , Yang J , Bruystens J , Cheung N , et al. Realizing the allosteric potential of the tetrameric protein kinase A RIα holoenzyme. Structure. 2011;19:265–276. 10.1016/j.str.2010.12.005 21300294 PMC3097484

[pro70332-bib-0009] Boras BW , Kornev A , Taylor SS , McCulloch AD . Using Markov state models to develop a mechanistic understanding of protein kinase A regulatory subunit RIα activation in response to cAMP binding. J Biol Chem. 2014;289:30040–30051. 10.1074/jbc.M114.568907 25202018 PMC4208011

[pro70332-bib-0010] Brennan JP , Bardswell SC , Burgoyne JR , Fuller W , Schröder E , Wait R , et al. Oxidant‐induced activation of type I protein kinase A is mediated by RI subunit interprotein disulfide bond formation. J Biol Chem. 2006;281:21827–21836. 10.1074/jbc.M603952200 16754666

[pro70332-bib-0011] Brown SH , Wu J , Kim C , Alberto K , Taylor SS . Novel isoform‐specific interfaces revealed by PKA RIIbeta holoenzyme structures. J Mol Biol. 2009;393:1070–1082. 10.1016/j.jmb.2009.09.014 19748511 PMC3435109

[pro70332-bib-0012] Bruystens JG , Wu J , Fortezzo A , Kornev AP , Blumenthal DK , Taylor SS . PKA RIα homodimer structure reveals an intermolecular interface with implications for cooperative cAMP binding and Carney complex disease. Structure. 2014;22:59–69. 10.1016/j.str.2013.10.012 24316401 PMC3963464

[pro70332-bib-0013] Bubis J , Vedvick TS , Taylor SS . Antiparallel alignment of the two protomers of the regulatory subunit dimer of cAMP‐dependent protein kinase I. J Biol Chem. 1987;262:14961–14966. 3667618

[pro70332-bib-0014] Burgers PP , Bruystens J , Burnley RJ , Nikolaev VO , Keshwani M , Wu J , et al. Structure of smAKAP and its regulation by PKA‐mediated phosphorylation. FEBS J. 2016;283:2132–2148. 10.1111/febs.13726 27028580 PMC4980077

[pro70332-bib-0015] Burgers PP , Ma Y , Margarucci L , Mackey M , van der Heyden MA , Ellisman M , et al. A small novel A‐kinase anchoring protein (AKAP) that localizes specifically protein kinase A‐regulatory subunit I (PKA‐RI) to the plasma membrane. J Biol Chem. 2012;287:43789–43797. 10.1074/jbc.M112.395970 23115245 PMC3527963

[pro70332-bib-0016] Cadd G , McKnight GS . Distinct patterns of cAMP‐dependent protein kinase gene expression in mouse brain. Neuron. 1989;3:71–79. 10.1016/0896-6273(89)90116-5 2619996

[pro70332-bib-0017] Cànaves JM , Leon DA , Taylor SS . Consequences of cAMP‐binding site mutations on the structural stability of the type I regulatory subunit of cAMP‐dependent protein kinase. Biochemistry. 2000;39:15022–15031. 10.1021/bi001563q 11106480

[pro70332-bib-0018] Canaves JM , Taylor SS . Classification and phylogenetic analysis of the cAMP‐dependent protein kinase regulatory subunit family. J Mol Evol. 2002;54:17–29. 10.1007/s00239-001-0013-1 11734894

[pro70332-bib-0019] Cao B , Lu TW , Martinez Fiesco JA , Tomasini M , Fan L , Simon SM , et al. Structures of the PKA RIα holoenzyme with the FLHCC driver J‐PKAcα or wild‐type PKAcα. Structure. 2019;27:816–828.e4. 10.1016/j.str.2019.03.001 30905674 PMC6506387

[pro70332-bib-0020] Chau AK , Bracken K , Bai L , Pham D , Good LL , Maillard RA . Conformational changes in protein kinase A along its activation cycle are rooted in the folding energetics of cyclic‐nucleotide binding domains. J Biol Chem. 2023;299:104790. 10.1016/j.jbc.2023.104790 37150322 PMC10279917

[pro70332-bib-0021] Erlichman J , Rosenfeld R , Rosen OM . Phosphorylation of a cyclic adenosine 3′:5′‐monophosphate‐dependent protein kinase from bovine cardiac muscle. J Biol Chem. 1974;249:5000–5003. 4367815

[pro70332-bib-0022] First EA , Taylor SS . Induced interchain disulfide bonding in cAMP‐dependent protein kinase II. J Biol Chem. 1984;259:4011–4014. 6323450

[pro70332-bib-0023] Hao Y , England JP , Bellucci L , Paci E , Hodges HC , Taylor SS , et al. Activation of PKA via asymmetric allosteric coupling of structurally conserved cyclic nucleotide binding domains. Nat Commun. 2019;10:3984. 10.1038/s41467-019-11930-2 31484930 PMC6726620

[pro70332-bib-0024] Haushalter KJ , Schilling JM , Song Y , Sastri M , Perkins GA , Strack S , et al. Cardiac ischemia‐reperfusion injury induces ROS‐dependent loss of PKA regulatory subunit RIα. Am J Physiol Heart Circ Physiol. 2019;317:H1231–H1242. 10.1152/ajpheart.00237.2019 31674811 PMC6962616

[pro70332-bib-0025] Herberg FW , Doyle ML , Cox S , Taylor SS . Dissection of the nucleotide and metal‐phosphate binding sites in cAMP‐dependent protein kinase. Biochemistry. 1999;38:6352–6360. 10.1021/bi982672w 10320366

[pro70332-bib-0026] Huang LJ , Durick K , Weiner JA , Chun J , Taylor SS . Identification of a novel protein kinase A anchoring protein that binds both type I and type II regulatory subunits. J Biol Chem. 1997;272:8057–8064. 10.1074/jbc.272.12.8057 9065479

[pro70332-bib-0027] Ilouz R , Bubis J , Wu J , Yim YY , Deal MS , Kornev AP , et al. Localization and quaternary structure of the PKA RIβ holoenzyme. Proc Natl Acad Sci U S A. 2012;109:12443–12448. 10.1073/pnas.1209538109 22797896 PMC3411989

[pro70332-bib-0028] Ilouz R , Lev‐Ram V , Bushong EA , Stiles TL , Friedmann‐Morvinski D , Douglas C , et al. Isoform‐specific subcellular localization and function of protein kinase A identified by mosaic imaging of mouse brain. eLife. 2017;6:e17681. 10.7554/eLife.17681 28079521 PMC5300705

[pro70332-bib-0029] Kannan N , Wu J , Anand GS , Yooseph S , Neuwald AF , Venter JC , et al. Evolution of allostery in the cyclic nucleotide binding module. Genome Biol. 2007;8:R264. 10.1186/gb-2007-8-12-r264 18076763 PMC2246266

[pro70332-bib-0030] Kim C , Cheng CY , Saldanha SA , Taylor SS . PKA‐I holoenzyme structure reveals a mechanism for cAMP‐dependent activation. Cell. 2007;130:1032–1043. 10.1016/j.cell.2007.07.018 17889648

[pro70332-bib-0031] Kim C , Xuong NH , Taylor SS . Crystal structure of a complex between the catalytic and regulatory (RIalpha) subunits of PKA. Science. 2005;307:690–696. 10.1126/science.1104607 15692043

[pro70332-bib-0032] Kinderman FS , Kim C , von Daake S , Ma Y , Pham BQ , Spraggon G , et al. A dynamic mechanism for AKAP binding to RII isoforms of cAMP‐dependent protein kinase. Mol Cell. 2006;24:397–408. 10.1016/j.molcel.2006.09.015 17081990 PMC1855097

[pro70332-bib-0033] León DA , Herberg FW , Banky P , Taylor SS . A stable alpha‐helical domain at the N terminus of the RIalpha subunits of cAMP‐dependent protein kinase is a novel dimerization/docking motif. J Biol Chem. 1997;272:28431–28437. 10.1074/jbc.272.45.28431 9353302

[pro70332-bib-0034] Liebschner D , Afonine PV , Baker ML , Bunkóczi G , Chen VB , Croll TI , et al. Macromolecular structure determination using X‐rays, neutrons and electrons: recent developments in Phenix. Acta Crystallogr D Struct Biol. 2019;75:861–877. 10.1107/S2059798319011471 31588918 PMC6778852

[pro70332-bib-0035] Lu TW , Wu J , Aoto PC , Weng JH , Ahuja LG , Sun N , et al. Two PKA RIα holoenzyme states define ATP as an isoform‐specific orthosteric inhibitor that competes with the allosteric activator, cAMP. Proc Natl Acad Sci U S A. 2019;116:16347–16356. 10.1073/pnas.1906036116 31363049 PMC6697891

[pro70332-bib-0036] Marbach F , Stoyanov G , Erger F , Stratakis CA , Settas N , London E , et al. Variants in PRKAR1B cause a neurodevelopmental disorder with autism spectrum disorder, apraxia, and insensitivity to pain. Genet Med. 2021;23(8):1465–1473. 10.1038/s41436-021-01152-7 33833410 PMC8354857

[pro70332-bib-0037] McKay DB , Steitz TA . Structure of catabolite gene activator protein at 2.9 A resolution suggests binding to left‐handed B‐DNA. Nature. 1981;290:744–749. 10.1038/290744a0 6261152

[pro70332-bib-0038] Newlon MG , Roy M , Morikis D , Hausken ZE , Coghlan V , Scott JD , et al. The molecular basis for protein kinase A anchoring revealed by solution NMR. Nat Struct Biol. 1999;6:222–227. 10.1038/6663 10074940

[pro70332-bib-0039] Otwinowski Z , Minor W . Processing of X‐ray diffraction data collected in oscillation mode. Methods Enzymol. 1997;276:307–326. 10.1016/S0076-6879(97)76066-X 27754618

[pro70332-bib-0040] Pawson T , Scott JD . Protein phosphorylation in signaling—50 years and counting. Trends Biochem Sci. 2005;30:286–290. 10.1016/j.tibs.2005.04.013 15950870

[pro70332-bib-0041] Reimann EM , Walsh DA , Krebs EG . Purification and properties of rabbit skeletal muscle adenosine 3′,5′‐monophosphate‐dependent protein kinases. J Biol Chem. 1971;246:1986–1995. 4324558

[pro70332-bib-0042] Sarma GN , Kinderman FS , Kim C , von Daake S , Chen L , Wang BC , et al. Structure of D‐AKAP2:PKA RI complex: insights into AKAP specificity and selectivity. Structure. 2010;18:155–166. 10.1016/j.str.2009.12.012 20159461 PMC3090270

[pro70332-bib-0043] Su Y , Dostmann WR , Herberg FW , Durick K , Xuong NH , Ten Eyck L , et al. Regulatory subunit of protein kinase A: structure of deletion mutant with cAMP binding domains. Science. 1995;269:807–813. 10.1126/science.7638597 7638597

[pro70332-bib-0044] Taylor SS , Herberg FW , Veglia G , Wu J . Edmond Fischer's kinase legacy: history of the protein kinase inhibitor and protein kinase A. IUBMB Life. 2023;75:311–323. 10.1002/iub.2714 36855225 PMC10050139

[pro70332-bib-0045] Taylor SS , Ilouz R , Zhang P , Kornev AP . Assembly of allosteric macromolecular switches: lessons from PKA. Nat Rev Mol Cell Biol. 2012;13:646–658. 10.1038/nrm3432 22992589 PMC3985763

[pro70332-bib-0046] Walsh DA , Ashby CD , Gonzalez C , Calkins D , Fischer EH , Krebs EG . Purification and characterization of a protein inhibitor of adenosine 3′,5′‐monophosphate‐dependent protein kinases. J Biol Chem. 1971;246:1977–1985. 4324557

[pro70332-bib-0047] Walsh DA , Perkins JP , Krebs EG . An adenosine 3′,5′‐monophosphate‐dependant protein kinase from rabbit skeletal muscle. J Biol Chem. 1968;243:3763–3765. 4298072

[pro70332-bib-0048] Wu J , Brown SH , von Daake S , Taylor SS . PKA type IIalpha holoenzyme reveals a combinatorial strategy for isoform diversity. Science. 2007;318:274–279. 10.1126/science.1146447 17932298 PMC4036697

[pro70332-bib-0049] Zhang P , Smith‐Nguyen EV , Keshwani MM , Deal MS , Kornev AP , Taylor SS . Structure and allostery of the PKA RIIβ tetrameric holoenzyme. Science. 2012;335:712–716. 10.1126/science.1213979 22323819 PMC3985767

[pro70332-bib-0050] Zheng J , Trafny EA , Knighton DR , Xuong NH , Taylor SS , Ten Eyck LF , et al. 2.2 A refined crystal structure of the catalytic subunit of cAMP‐dependent protein kinase complexed with MnATP and a peptide inhibitor. Acta Crystallogr D Biol Crystallogr. 1993;49:362–365. 10.1107/S0907444993000423 15299527

