# Supplementary figures and images for: N3A motifs in RIβ mediate allosteric crosstalk between cAMP and ATP in PKA activation
Source: Protein Sci. 2025 Oct 18;34(11):e70332. doi: 10.1002/pro.70332 (PMC12535202; doi:10.1002/pro.70332)

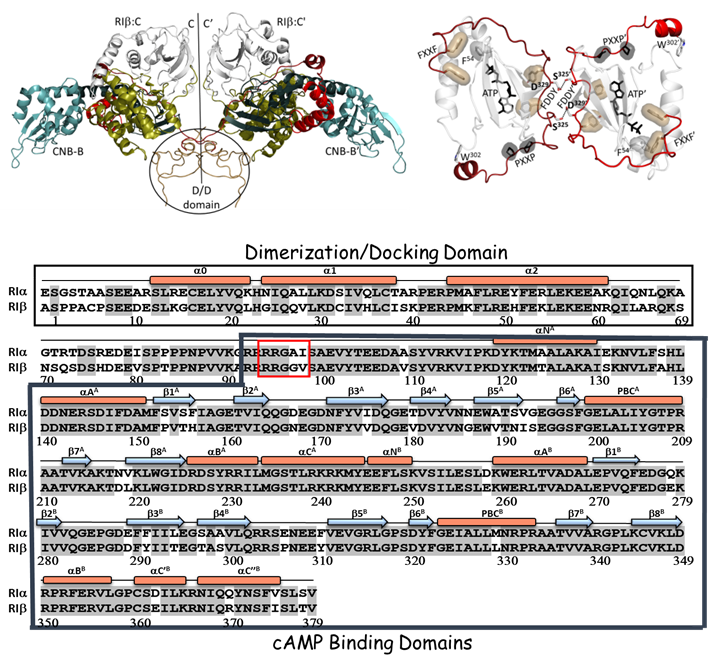

Supplement: Supplementary file 1 — Figure S1. Crystal structure of RIβ2C2 holoenzyme. (top left) The structure of RIβ2C2 holoenzyme with the D/D domain visible (black circle). (top right) The anti‐parallel dimer interface between the C‐tails of the two C‐subunits. (bottom) Sequence alignment of human RIα and RIβ. The secondary structure elements are shown in cartoon. [file PRO-34-e70332-s008.tif]

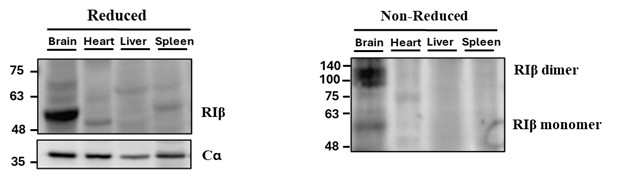

Supplement: Supplementary file 2 — Figure S2. Protein expression and dimerization of RIβ in mouse tissues. Western blot analysis of protein samples from brain, heart, liver, and spleen of mice were performed under reduced and non‐reduced conditions. (left, reduced conditions): Protein expression of RIβ was performed in reduced conditions, showing the monomeric form of the protein (~50 kDa) in all tissues, along with PKA catalytic subunit Cα. (right, non‐reduced conditions): RIβ is dimer in non‐reduced conditions, showing the dimeric form (~100 kDa) in brain. The blot was probed with the RIβ specific antibody. [file PRO-34-e70332-s005.tif]

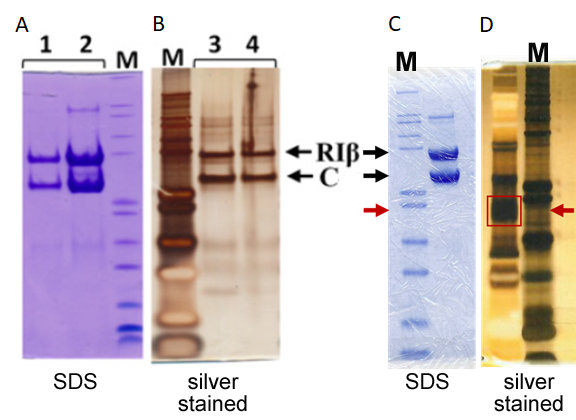

Supplement: Supplementary file 3 — Figure S3. Gel electrophoresis of the crystal confirmed cleavage of the full‐length RIβ. (a) The full‐length RIβ2C2 holoenzyme used for crystallization, as shown previously in Figure S2 (Ilouz et al. 2012). (b) The diffracting crystals contain the full‐length RIβ2C2 holoenzyme, as was also shown previously in Figure S2 (Ilouz et al. 2012). (c) Following purification of the RIβ2C2 holoenzyme, no degradation products were observed even after storage of the holoenzyme for 2 weeks at 4°C. (d) The diffracting crystal shows the RIβ had been degraded to a 38 kDa fragment, as shown by the red arrow and square. Panels (a, c) are SDS gels; panels (b, d) are silver stained gels. [file PRO-34-e70332-s001.tif]

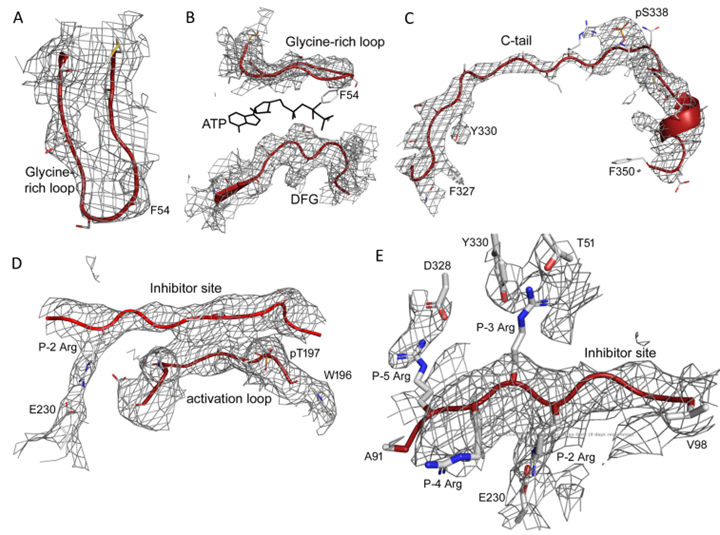

Supplement: Supplementary file 4 — Figure S4. Density maps of motifs in the kinase domain. (a) Top view of the G‐loop, 2Fo‐Fc density at 1σ are in gray. (b) Side view of the G‐loop and DFG motif, ATP (black) is from 1ATP when superimposed with the C‐subunit of RIβ:C structure. There is no density for ATP. (c) C‐tail of C‐subunit. The side chains of some key residues are also shown. (d) The Inhibitor site of RIβ and the Activation loop of C‐subunit. The P‐2 Arg interacts with E230, their density are well traced. (e) The Inhibitor site and FDDY motif. P‐3 Arg is flexible as its side‐chain density is not traced. [file PRO-34-e70332-s011.tif]

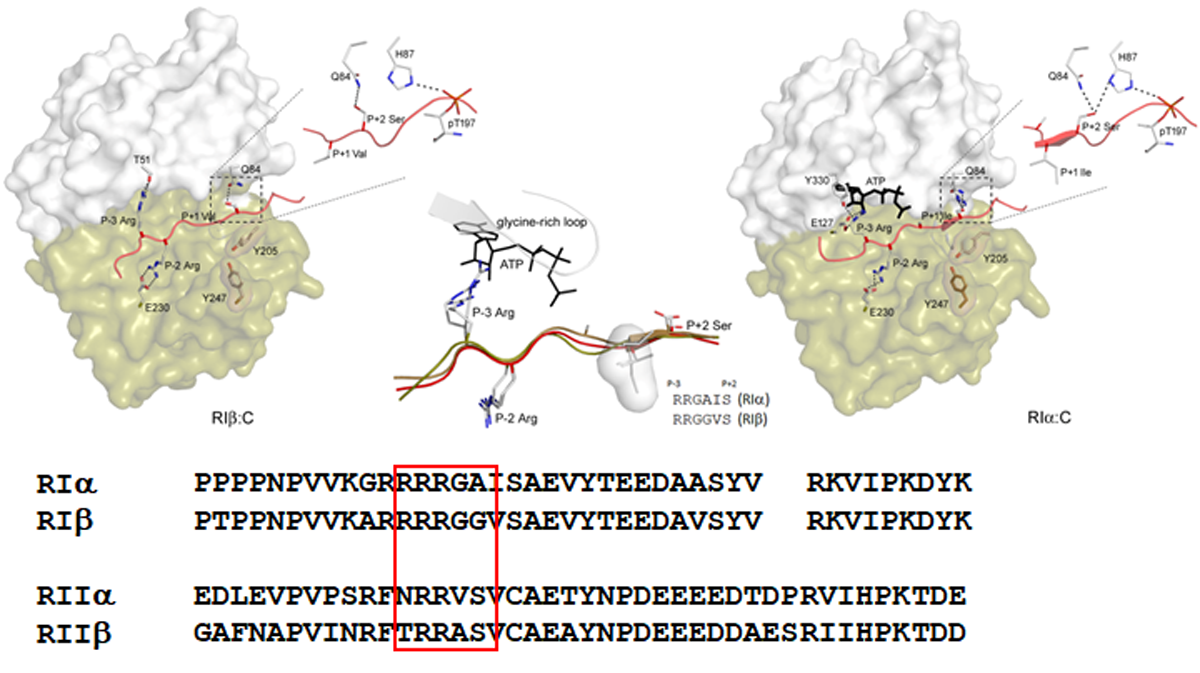

Supplement: Supplementary file 5 — Figure S5. Inhibitor sites are different in RIα and RIβ. (left) Inhibitor site of RIβ docks into the active site cleft of C‐subunit. ATP is missing. (right) Inhibitor site of RIα docks into the active site cleft of C‐subunit. ATP is in black stick. (inset) The structural comparison of two Inhibitor sites. (bottom) The sequence alignment of the Inhibitor sites of four R‐subunit isoforms. [file PRO-34-e70332-s002.tif]

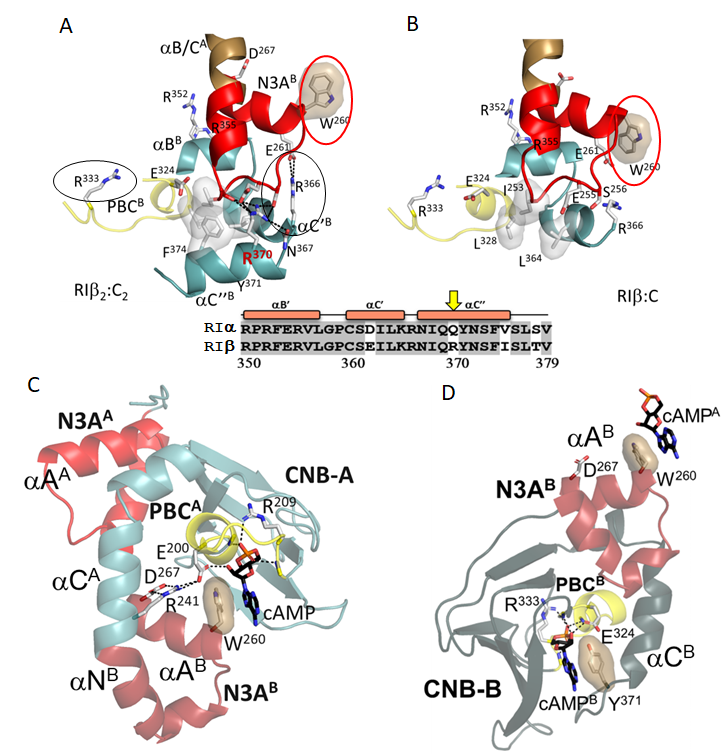

Supplement: Supplementary file 6 — Figure S6. W260 plays important functional role. (a) N3AB motif in RIβ2C2 structure. The hydrophobic surface among N3AB (red), PBCB (yellow) and C‐tail (light blue) are shown as white shell. W260 is circled in red. (b) N3AB motif in RIβ:C structure, the same view as (a). N3AB sequence alignment in RIα and RIβ is also shown. (c) W260 is the cAMP capping residue of CNB‐A in the cAMP bound RIα. The N3AA is colored in red and N3AB in dark red. (d) Y371, from the C″‐helix, is the cAMP capping residue of CNB‐B in the cAMP bound RIα. W260 and cAMP in CNB‐A are also shown. [file PRO-34-e70332-s004.tif]

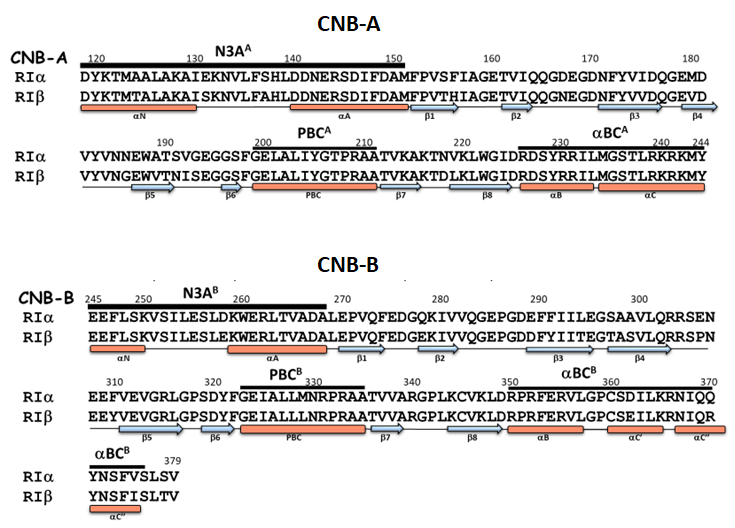

Supplement: Supplementary file 7 — Figure S7. The sequence alignment of CNB‐A (top) and CNB‐B (bottom) in RIα and RIβ. The secondary structure elements are shown in cartoon. [file PRO-34-e70332-s010.tif]

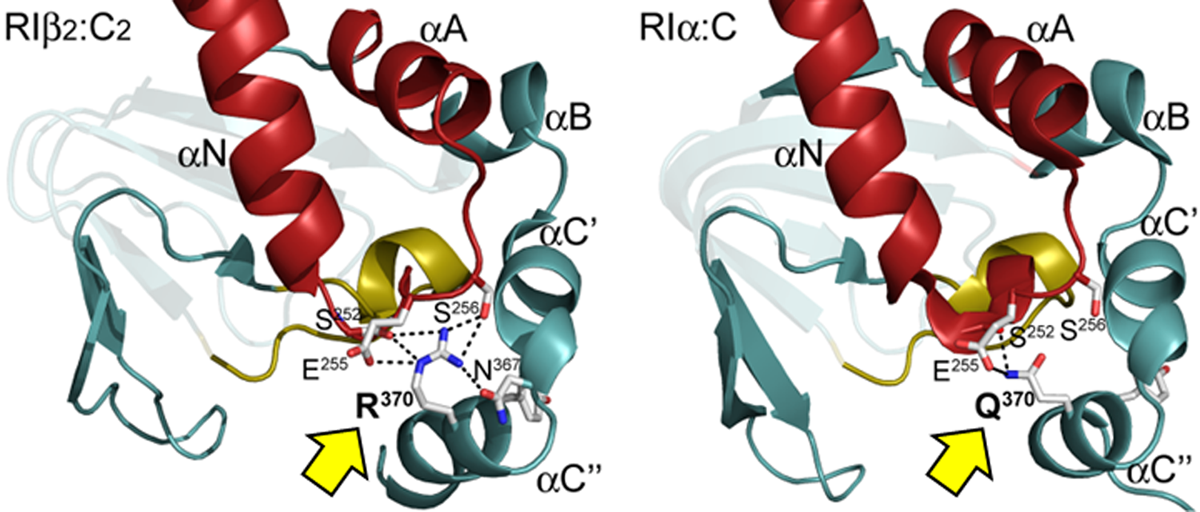

Supplement: Supplementary file 8 — Figure S8. R370 is a key functional residue in RIβ. (left) R370 interacts with several residues in the 310 loop of N3AB. (right) Q370 has much weaker interaction with N3AB in RIα. [file PRO-34-e70332-s006.tif]

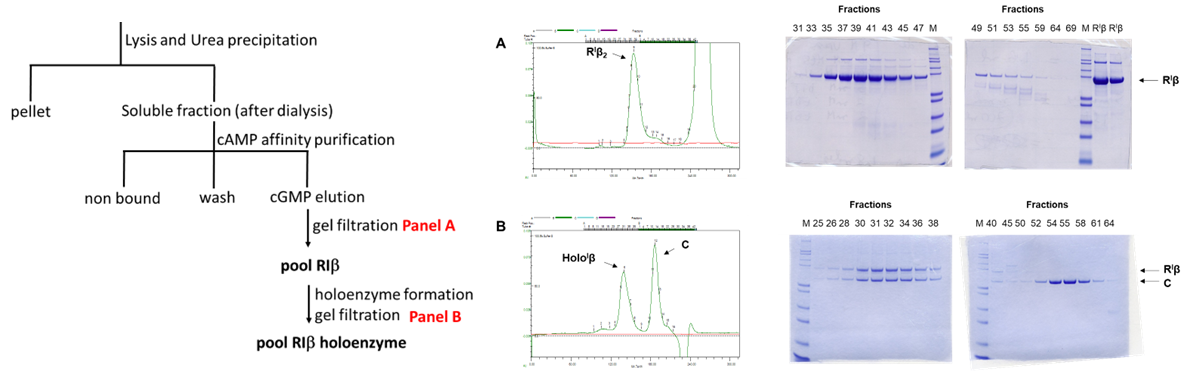

Supplement: Supplementary file 9 — Figure S9. Purification of the RIβ through a gel filtration column. (left) The protocol for purification of RIβ. (right) Panel (a) shows the purification of the cGMP‐eluted RIβ. As seen in the gel (top right), there are some degradation products. Panel (b) shows the RIβ holoenzyme isolated on the same gel filtration column. As seen in the gels (bottom right), no breakdown products were observed. This holoenzyme was routinely pooled and stored at 4°C for many weeks; no degradation was observed (Figure S3c). [file PRO-34-e70332-s009.tif]

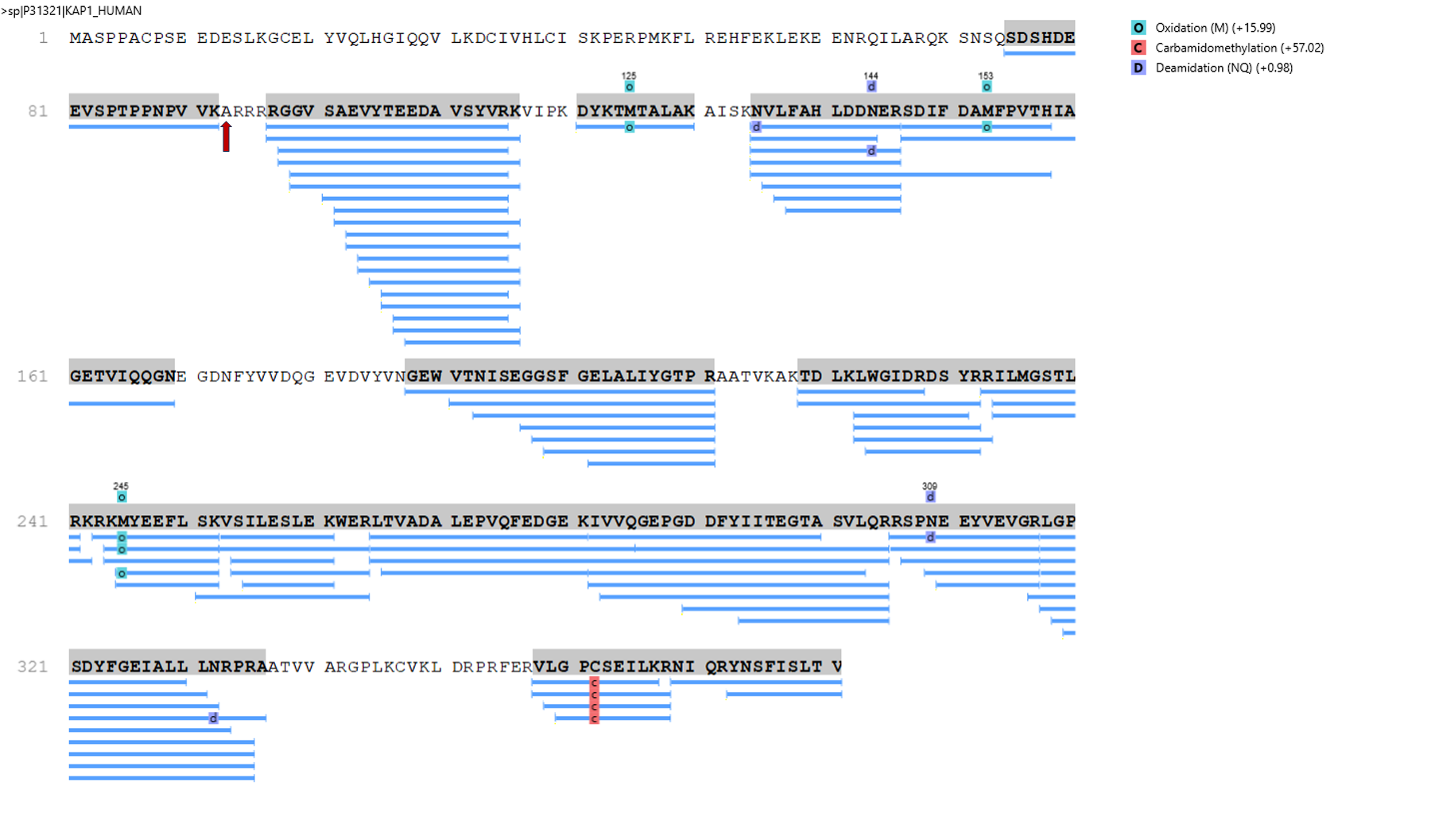

Supplement: Supplementary file 10 — Figure S10. Mass spectrometry analysis of a 38 kDa cleavage product. The 38 kDa band from a stored sample of RIβ, one of the most abundant protein species in the analysis, was excised from a SDS gel and analyzed by mass spectrometry. The results confirm this sample was a degradation of the full‐length protein with most of the N‐terminus missing. The red arrow shows the first traceable residue in the RIβ:C structure. [file PRO-34-e70332-s012.tif]
