# Supplementary material for: N3A motifs in RIβ mediate allosteric crosstalk between cAMP and ATP in PKA activation
Source: Protein Sci. 2025 Oct 18;34(11):e70332. doi: 10.1002/pro.70332 (PMC12535202; doi:10.1002/pro.70332)
Supplement: Supplementary file 11 — Table S1. Data collection and refinement statistics. [file PRO-34-e70332-s003.doc]

**Table 1:** **Data Collection and Refinement Statistics**

|  | RIβ:C |
| --- | --- |
| **Data collection** |  |
| Space group | P6522 |
| Cell dimensions (Å)  *a=b*  *c* | 103.5  313.4 |
| No. of molecule per asymmetrical unit | 1 |
| Resolution (Å) | 3.7 |
| *R*_merge_ | 0.069 (0.47) |
| Completeness (%) | 99.5 (99.0) |
| I/sigma | 30.8 (8.4) |
| No. reflections | 11338 |
|  |  |
| **Refinement** |  |
| Resolution (Å) | 50.0-3.7 |
| *R*_work_ / *R*_free_ (%) | 23.1/29.3 |
| No. of protein residues | 638 |
| R.m.s. deviations |  |
| Bond lengths (Å) | 0.013 |
| Bond angles (°) | 2.0 |
| Ramachandran angles (%) |  |
| most favored | 93.0 |
| disallowed | none |

*Values in parentheses are for highest-resolution shell: (3.70-3.78 Å)
