# Supplementary material for: N3A motifs in RIβ mediate allosteric crosstalk between cAMP and ATP in PKA activation
Source: Protein Sci. 2025 Oct 18;34(11):e70332. doi: 10.1002/pro.70332 (PMC12535202; doi:10.1002/pro.70332)
Supplement: Supplementary file 12 — Table S2. Residue contacts in RIβ in the Holo (H) and cAMP bound (B) conformation. [file PRO-34-e70332-s007.docx]

**Table 2: Residue Contacts in RIβ in the Holo (H) and cAMP bound (B) conformation.**

Residue Number H-Conformation (RIβ/RIα) B-Conformation (RIα)

E200 N133; N of L135 (3^10^ loop) cAMP^A^ and NH1/NH2 of R241

Y205 Y247 of C-subunit Solvent exposed

R241 K213 CO of C-subunit, D267 E200 and D267

K242 E246 Solvent exposed

E245 Solvent exposed K259

K250 Solvent exposed, CO of E246 Solvent exposed

V251 Packs with PBC (F290/I253) Packs with I292 and Y321

S252 R370 (αC”) Solvent exposed

E255 R370 (αC”) Solvent exposed

S256 R370 (αC”) Solvent exposed

D258 Capping αA^B^ Capping αA^B^

K259 Solvent exposed E245

W260 Packs with K192 of C-subunit capping cAMP^A^

E261 R366 N of D258

R262 Solvent exposed CO of L254 and L257

E267 R194 of C-subunit, R241 R241

R331 Solvent exposed; CO to G287 Solvent exposed

R333 Solvent exposed cAMP^B^-PO3; CO of G323 and E289

R366 E261 Solvent exposed

**R370 (RIβ/R_2_C_2_) S252, E255, S256 (3^10^ loop)**

**R370 (RIβ/R:C) Flexible/Solvent exposed**

**Q370 (RIα) S252, E255 (3^10^ loop) Solvent exposed**
